# Evolution of a New Function by Degenerative Mutation in Cephalochordate Steroid Receptors

**DOI:** 10.1371/journal.pgen.1000191

**Published:** 2008-09-12

**Authors:** Jamie T. Bridgham, Justine E. Brown, Adriana Rodríguez-Marí, Julian M. Catchen, Joseph W. Thornton

**Affiliations:** 1 Center for Ecology and Evolutionary Biology, University of Oregon, Eugene, Oregon, United States of America; 2 Institute of Neuroscience, University of Oregon, Eugene, Oregon, United States of America; 3 Department of Computer and Information Science, University of Oregon, Eugene, Oregon, United States of America; Fred Hutchinson Cancer Research Center, United States of America

## Abstract

Gene duplication is the predominant mechanism for the evolution of new genes. Major existing models of this process assume that duplicate genes are redundant; degenerative mutations in one copy can therefore accumulate close to neutrally, usually leading to loss from the genome. When gene products dimerize or interact with other molecules for their functions, however, degenerative mutations in one copy may produce repressor alleles that inhibit the function of the other and are therefore exposed to selection. Here, we describe the evolution of a duplicate repressor by simple degenerative mutations in the steroid hormone receptors (SRs), a biologically crucial vertebrate gene family. We isolated and characterized the SRs of the cephalochordate *Branchiostoma floridae*, which diverged from other chordates just after duplication of the ancestral SR. The *B. floridae* genome contains two SRs: BfER, an ortholog of the vertebrate estrogen receptors, and BfSR, an ortholog of the vertebrate receptors for androgens, progestins, and corticosteroids. BfSR is specifically activated by estrogens and recognizes estrogen response elements (EREs) in DNA; BfER does not activate transcription in response to steroid hormones but binds EREs, where it competitively represses BfSR. The two genes are partially coexpressed, particularly in ovary and testis, suggesting an ancient role in germ cell development. These results corroborate previous findings that the ancestral steroid receptor was estrogen-sensitive and indicate that, after duplication, BfSR retained the ancestral function, while BfER evolved the capacity to negatively regulate BfSR. Either of two historical mutations that occurred during BfER evolution is sufficient to generate a competitive repressor. Our findings suggest that after duplication of genes whose functions depend on specific molecular interactions, high-probability degenerative mutations can yield novel functions, which are then exposed to positive or negative selection; in either case, the probability of neofunctionalization relative to gene loss is increased compared to existing models.

## Author Summary

Most genes evolved by duplication of more ancient genes. Under existing models of this process, mutations that compromise one copy have no effect on the other; as long as one copy remains intact, such “degenerative” mutations are shielded from selection. Because degenerative mutations are common, most duplicates are expected to be disabled before new functions can evolve. The great functional diversity of genes is therefore somewhat puzzling. Here, we reconstruct how simple degenerative mutations produced a new function in the steroid hormone receptors (SRs), a gene family crucial to reproduction and development. We characterized the two SRs of *B. floridae*, a cephalochordate that diverged from vertebrates ∼500 million years ago, just after the ancestral SR duplicated. One retained the ancestral gene's estrogen receptor–like functions, while the other evolved a new function as a competitive repressor of the first. Either of two historical mutations is sufficient to recapitulate evolution of this function by disabling the receptor's response to estrogen, but leaving its DNA-binding capacity intact. Our results suggest that, for the many genes that function by specifically interacting with other molecules, simple mutations can yield novel functions that, beneficial or deleterious, are exposed to selection.

## Introduction

The vast majority of genes in eukaryotic genomes are hierarchically organized in gene families and superfamilies, because they were generated by a serial process of gene duplication and divergence [1],[2]. The complexity of gene regulatory networks is also due to this process, which produced new functional interactions between regulators, effectors, and target genes [3],[4]. How genes and their functions evolve after duplication is therefore a central and long-standing question in evolutionary biology [5]–[10].

### Gene Duplication Models

All major models of duplicate gene evolution to date assume that the two genes' products do not interact with each other physically or functionally. Mutations in one copy therefore have no effect on the functions of the other.

**Figure 1:**
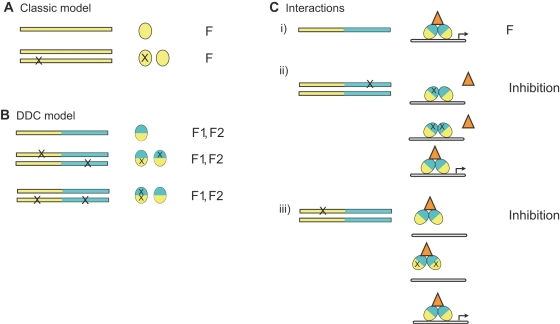
Effect of degenerative mutations on the function of a duplicate gene that depends on molecular interactions. A) In the classic model, duplicate genes are redundant and do not interact, so mutation in one copy causes no change in the function (F) and evolve neutrally. B) In the DDC model, duplicate genes, which have two or more modular subfunctions (F1 and F2, blue and yellow), are redundant and do not interact. Degenerative mutations in subfunctions cause no change in function if the subfunction is retained in the other copy and evolve neutrally. C) If a gene depends upon molecular interactions for its function, degenerative mutations in one copy can affect the function of the other and therefore be selected for or against. i) In the case shown, a gene product must dimerize and interact with DNA (white bar) and an accessory factor (orange triangle) for its function (transcriptional activation). ii) After duplication, degenerative mutations that impair the interactions of one copy with the accessory factor yield a nonfunctional product that competes for DNA binding sites, reducing the activity of the other copy. iii) Degenerative mutations that impair DNA binding in one copy yield nonfunctional products that compete for accessory factors and reduce the activity of the other copy. Inhibition also occurs if the duplicate genes do not dimerize but compete for other binding partners.

In the classic model [7],[11], having two copies of a gene is phenotypically equivalent to having only one. After duplication, one copy drifts neutrally, free to amass mutations without the constraints of purifying selection (Figure 1A). In the vast majority of cases, degenerative mutations cause one copy to irreversibly lose its function and ultimately disappear from the genome, a process called nonfunctionalization. Rarely, however, mutations that yield a novel, beneficial function occur by chance; selection fixes these mutations and subsequently maintains both the original and “neofunctionalized” copies in the genome. The ultimate fate of a duplicate gene therefore depends on the outcome of a race between neo- and nonfunctionalization. Because gain-of-function mutations are very rare compared to those that compromise function [12]–[15], the vast majority of drifting duplicate genes will be lost, typically within a few million years, long before new functions are expected to evolve [10]. The plausibility of the classic model as a general explanation for the evolution of new functions has therefore been called into question[16],[17].

In the second model–Duplication, Degeneration and Complementation (DDC) [18]–duplication of a gene with multiple independent subfunctions, such as different expression domains controlled by separate regulatory elements, can be followed by degenerative mutations that knock out different subfunctions in each copy. Because the two copies complement each other but do not interact, these high-probability changes can occur neutrally in small populations, and purifying selection will subsequently conserve both copies and their remaining subfunctions (Figure 1B). The DDC model appears applicable in numerous case studies [18]–[21] and may explain in part why many duplicate genes bear the signature of continuing purifying selection after duplication [22]. Although subfunctionalization does not preclude subsequent evolution of new functions [23],[24], the DDC model per se does not explain how or why novel gene functions evolve after duplication, as has been observed in numerous examples [25]–[36].

In the third model, an increase in the dose of a gene's product due to duplication yields an immediate selective advantage; thereafter, purifying selection purges degenerative mutations and conserves the ancestral function in both copies[37]–[39]. This model says nothing about how gene pairs with different functions evolved, and it does not explain the long-term retention of duplicate genes for which dosage is not likely to be limiting for fitness, such as many signaling molecules and enzymes in multicellular eukaryotes. Other models have also been suggested, largely variants of the three major models [16], [40]–[43].

### Molecular Interactions and Gene Duplication

Given the high probability of degenerative mutations relative to gain-of-function mutations, it remains unclear how large numbers of duplicate genes have evaded nonfunctionalization long enough to evolve new functions. A key omission from the major models is their assumption that the products of duplicate genes do not interact, either through direct physical contact or by competing for other molecular partners. Gene duplication is recognized as providing raw material for the evolution of elaborate molecular interaction networks [3],[44], but only limited research has addressed how interactions affect the evolutionary fate of duplicate genes. Most of this work has focused on the possibility that duplications may alter the stoichiometry of proteins in a complex, resulting in selection for or against the duplication per se [44]–[46].

But molecular interactions may affect the fate of gene duplicates much more directly, and two types are of particular interest (Figure 1C). First, many proteins function as homodimers. After duplication of such genes, their products will initially cross-dimerize [44]. Even if the duplicate genes are functionally redundant–in the sense that two copies produce the same phenotype as one–mutations that compromise one duplicate may interfere with the functionality of the other by tying up its products in non-functional dimers, just as null mutations at a single locus can produce dominant negative alleles (see refs. [8],[47]). Second, many gene products form functional complexes with other molecules, such as other proteins, DNA binding sites, or small-molecule ligands and substrates. After duplication, the two genes' products initially compete for the same binding partners [44]; mutations in one copy that compromise function but not binding will tie up partner molecules in nonfunctional complexes, reducing the activity of the other copy. As a result, degenerative mutations after duplication may not be phenotypically silent, even when the other copy retains the ancestral alleles. If repression of the ancestral function is deleterious, as seems likely in most cases, then purifying selection will tend to remove degenerative mutations from both copies of a gene. As a result, the temporal window before nonfunctionalization will be longer than expected under the neutral scenarios of the classic and DDC models, and the relative probability of neofunctionalization will increase. More rarely, the evolution of a repressor molecule may allow beneficial new modes of gene regulation; selection would favor such mutations, and a neofunctionalization will occur by high-probability degenerative mutations rather than the low-probability events required under the other models.

### Steroid Hormone Receptors

Steroid hormone receptors (SRs) exemplify both types of functional interaction described above [48],[49], and their evolution has been the subject of considerable interest [50]–[57]. SRs are the major mediators of the effects of gonadal and adrenal steroid hormones on development, reproduction, behavior, and homeostasis throughout the vertebrates. These ligand-controlled transcription factors bind tightly as homodimers to specific DNA response elements in the control region of target genes. Jawed vertebrates have six SRs–two for estrogens (ERα and ERβ) and one each for testosterone and other androgens (AR), progestins (PR), glucocorticoids (GR), and mineralocorticoids (MR)–although some lineages, such as the teleosts, have additional duplicates of these genes [55],[57],[58]. Each hormone binds with high affinity and specificity to a receptor, triggering a change in the receptor's conformation that allows it to attract coactivator proteins that facilitate transcription of the target gene. SRs have a modular structure, including a highly conserved DNA-binding domain (DBD)–which recognizes and binds to response elements–and a moderately conserved ligand-binding domain (LBD), which binds the hormone and contains the hormone-activated transcriptional activation function. This modular structure allows, for example, construction of chimeric proteins that combine the functions of one protein's DBD with those of another protein's LBD [59], and many mutations are known that independently compromise ligand binding, DNA binding, or transcriptional activation, without impairing the other functions [60]–[65].

The SR gene family evolved by duplication and acquisition of novel functions from a single ancestral SR gene (AncSR1). AncSR1 is as ancient as the protostome-deuterostome divergence, based on discovery of a steroid receptor gene in mollusks [27],[66]. Duplication of this ancestral gene produced two major subfamilies of vertebrate SRs[29]. One contains the estrogen receptors ERα and ERβ, which are activated by estrogens and bind to estrogen-response elements (EREs, inverted palindromes of AGGTCA) [67]. Members of the other subfamily–the ketosteroid receptors (kSRs), including AR, PR, GR, and MR–are activated by steroids with a keto group at the 3-position on the steroid backbone (in contrast to estrogens, which have a 3-hydroxyl) and bind to ketosteroid response elements (kSREs, inverted palindromes of AGAACA) [67]. Phylogenetic reconstruction, gene synthesis, and experimental characterization of AncSR1 has shown that it had the ligand- and DNA-specificity of the vertebrate estrogen receptors [29],[66], indicating that the gene duplicate leading to the kSRs evolved novel DNA- and ligand-binding functions.

The evolutionary events by which this neofunctionalization process occurred are obscure. It was complete by the time of the vertebrate ancestor, some 470 million years ago, because agnathans, the most basal vertebrate taxon, contain one ortholog each for the ERα/ERβ, the GR/MR, and the AR/PR pairs of jawed vertebrates [29]. But little information is available on the interval between the AncSR1 duplication and the ancestral kSR, because SRs have not been characterized in taxa that branch off the animal phylogeny between the protostome-deuterostome ancestor and the ancestral vertebrate. Sequenced genomes are available from the echinoderm *Strongylocentrotus purpuratus* and the urochordate *Ciona intestinalis*, but all SRs were lost completely in these lineages [68],[69].

Cephalochordates therefore have the potential to provide key information about the early evolution of the kSR gene after duplication of AncSR1 [50]. These animals–commonly known as lancelets or amphioxus–represent the earliest-branching chordate taxon [70],[71]. In many gene families, cephalochordates contain a single gene orthologous to up to four paralogs in vertebrates [72], so they are a good candidate to have a single kSR ortholog. Further, the genome of the cephalochordate *Branchiostoma floridae* contains the set of cytochrome P450 (CYP) genes required for the biosynthesis of the sex steroids testosterone, progesterone, and estradiol, and the synthesis of these hormones in *B. floridae* has been experimentally established [73],[74]. To gain insight into the early functional diversification of the SRs, we therefore sought to isolate the SRs of *B. floridae*, characterize their functions, and analyze their evolution.

## Results

### Cephalochordate Steroid Receptor Sequences and Phylogenetic Analysis

**Figure 2:**
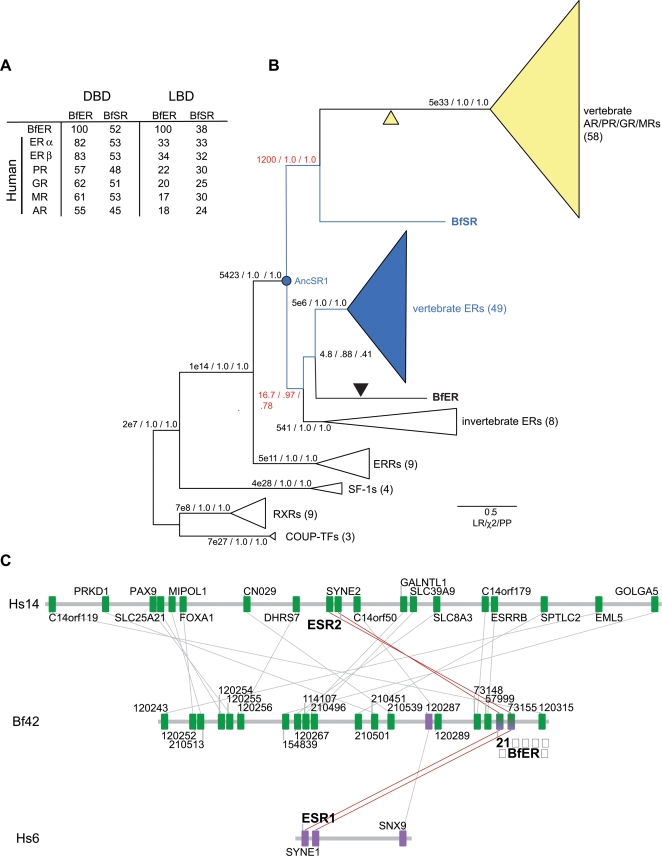
Cephalochordates have one ortholog each of the ER and kSR subfamilies. A) Sequence similarity of BfER and BfSR to each other and human steroid receptors. The percent of identical amino acid residues for each pairwise comparison is shown. DBD and LBD, DNA-binding and ligand-binding domains. B) Reduced phylogeny of the steroid receptor gene family. Numbers in parentheses refer to the number of sequences in each group used in the analysis. Estrogen-sensitive receptors, including the ancestral steroid receptor (AncSR1) are in blue; ketosteroid receptors are in yellow. Black triangle, loss of transcriptional activation; yellow triangle, gain of ketosteroid sensitivity in the vertebrate AR/PR/GR/MR clade. Support for each branch is shown as the approximate likelihood ratio (the ratio of the likelihood of the best tree with that node to the best tree without it), the chi-square likelihood confidence statistic for the node, and the Bayesian posterior probability. Nodes that place *B. floridae* receptors as orthologs of the ERs and kSRs are in red. Scale bar shows expected per-site substitution rate for branch lengths. Complete phylogenies and a list of genes, species, and accessions are in Figures S6, S7, S8 and Table S1. C) Conserved synteny between BfER-containing scaffold 42 and human chromosomes 14 and 6, which contain ERβ (ESR2) and ERα (ESR1), respectively. Green boxes with connecting lines indicate reciprocal BLAST best-hits between scaffold 42 in the *B. floridae* genome and human chromosome 14; purple boxes, orthologs between Bf scaffold 42 and human chromosome 6. Genes are shown in order with spacing approximately proportional to physical distance. BfER, ESR1, and ESR2 all share orthologous nearest neighbors (red lines).

Using a reciprocal BLAST search strategy, we identified two loci as steroid receptor orthologs in the completely sequenced genome of *B. floridae*. Coding sequences of both genes were determined using RACE (rapid amplification of cDNA ends) on RNA extracted from *B. floridae* adults, and full-length coding sequences were then isolated using the polymerase chain reaction. One of the *B. floridae* receptors has high amino acid identity to the human ERs, particularly in the DNA-binding domain, and much lower similarity to the AR, PR, GR, and MR. The other has approximately equal similarity to the ERs and the other SRs (Figure 2A, S1, S2).

To determine orthology relationships, we phylogenetically analyzed an alignment of the two *B. floridae* SRs with the protein sequences of 140 other steroid and related receptors (Table S1). Both maximum likelihood and Bayesian analysis place the ER-like *B. floridae* receptor at the base of the chordate estrogen receptor clade and the other receptor at the base of the kSR clade (Figure 2B). We therefore named the former gene BfER and the latter BfSR. The affinity of BfER with other deuterostome and protostome ERs and of the BfSR with the vertebrate kSRs are both well-supported, with high posterior probabilities and likelihood ratios (Figure 2B). Alternative topologies that place the BfER and BfSR as cephalochordate-specific duplicates are very unlikely, with a cumulative posterior probability <0.20 in the BMCMC analysis. Only the taxonomic relationships within the ERs are not well-resolved; we cannot rule out an alternative topology in which the protostome “ERs” are sister to a clade of all deuterostome ERs and kSRs, implying duplication of AncSR1 after the protostome-deuterostome divergence. The phylogeny we recovered is not an artifact of unincorporated heterotachy (site-specific changes in evolutionary rate), which under some conditions can confound ML and BMCMC methods using homogeneous models [75],[76]: when sequences were analyzed using a mixed branch-length method that incorporates heterotachy [77], the placement of BfER and BfSR did not change (See Figure S3).

This phylogeny indicates that BfER is orthologous to the ERα and ERβ pair of humans, and BfSR is orthologous to the AR/PR/GR/MR cluster. The duplication of AncSR1 to produce these two major SR clades therefore occurred before the divergence of cephalochordates from the lineage leading to vertebrates. Subsequent duplications to produce the four kSRs and ERs of vertebrates occurred after that split, likely in the two vertebrate-specific genome duplications [29],[78]. This scenario is consistent with evidence of conserved synteny between the scaffold containing BfER and human chromosomes 6 and 14, where ERα and ERβ are located (Figures 2C, S4, see also ref. [79]). The short scaffold that contains BfSR does not contain signal of strong synteny to any human chromosomes (Figure S4). The phylogenetic placement of the *B. floridae* receptors close to the ER-like AncSR1 explains why both have substantial sequence similarity to the human ERs. The relatively long branch subtending the AR/PR/GR/MR clade indicates a period of relatively rapid sequence evolution in the ancestral kSR in the lineage leading to the vertebrates after the cephalochordates diverged and before subsequent gene duplications.

### Intrinsic Functions of BfSR and BfER

**Figure 3:**
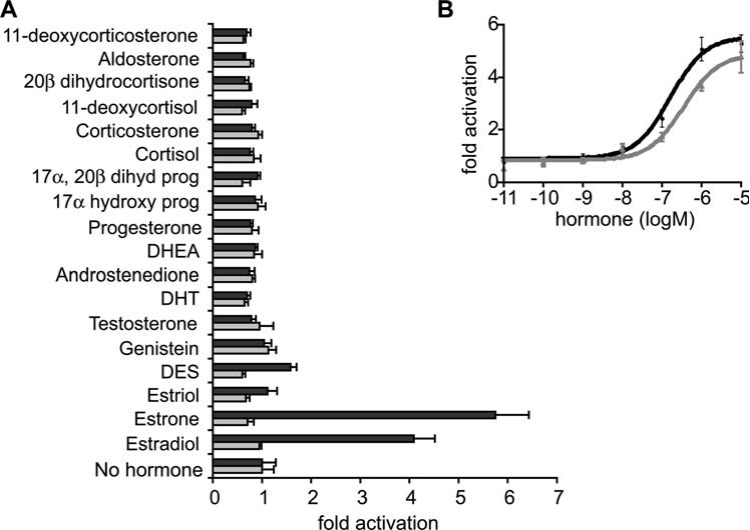
Estrogens activate BfSR but not BfER. A) Hormone-activated transcription of a luciferase reporter gene by the ligand-binding domains of BfSR (black) and BfER (gray) is shown as fold activation relative to vehicle-only control. All hormone treatments were at 1 µM concentration. DES, diethylstilbesterol; DHT, dihydrotestosterone; DHEA, dehydroepiandrosterone; Prog, progesterone. Mean+/−SE of three replicates is shown. B) Dose-response relationship for transcriptional activation by BfSR LBD in the presence of increasing concentrations of estradiol (black) and estrone (gray).

**Figure 4:**
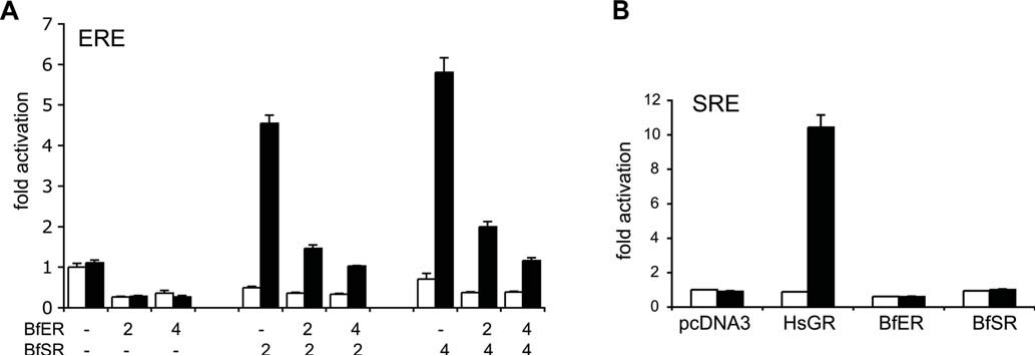
BfER represses BfSR-mediated transcription on estrogen response elements. A) Full-length BfSR activates transcription of an ERE-driven reporter gene in the presence of 1 µM estradiol (black bars), but activation is inhibited by transfection of increasing quantities of BfER. The quantity of each plasmid transfected is indicated in ng/well. White bars, vehicle-only (no hormone) control. B) Neither BfER nor BfSR activates reporter transcription from an SRE binding site. Cells were transfected with an SRE-driven luciferase reporter and full-length BfER, BfSR, empty vector (pcDNA3), or positive control (HsGR; human GR). Black bars, hormone treatment at 1 uM; white bars, vehicle only.

To determine the molecular functions of the *B. floridae* steroid receptors, we assayed both full-length proteins and specific functional domains in a cell culture system. Surprisingly, we found that BfSR retains the ER-like functions of the ancestral receptor, but BfER does not. BfSR's LBD activates transcription in the presence of nanomolar concentrations of estradiol and estrone, but is insensitive to ketosteroids, including a broad panel of androgens, progestins, and corticoids (Figures 3A, 3B). In the presence of estrogens, the full-length BfSR activates transcription of an ERE-driven reporter gene (Figures 4A, S5), but it does not activate genes driven by the SRE recognized by vertebrate kSRs (Figure 4B). These data indicate that the derived ligand-binding and DNA-recognition properties of the vertebrate kSRs evolved by neofunctionalization after the cephalochordate/vertebrate divergence.

**Figure 5:**
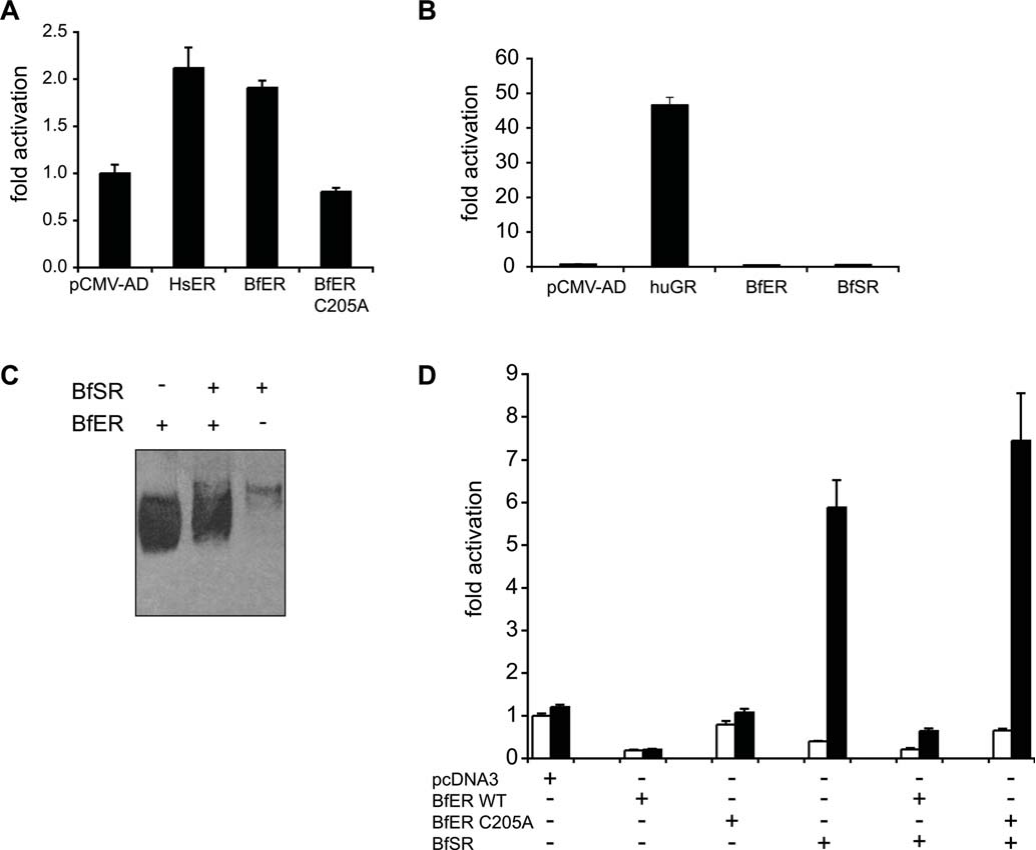
BfER repression of BfSR by competition for EREs. A) BfER's DNA-binding domain recognizes an estrogen response element (ERE). A fusion of the BfER-DBD with a constitutive activation domain activates transcription of an ERE-driven luciferase reporter gene. Negative control (pCMV-AD vector only) and positive control (HsER, human ERα DBD) are shown for comparison. Replacement C205A in the BfER-DBD abolishes recognition of an ERE. B) Neither the BfER-DBD nor the BfSR-DBD recognize steroid response elements (SRE). Negative control (pCMV-AD vector only) and positive control (HsGR, human GR DBD) are shown for comparison. C) BfER and BfSR bind directly to EREs. An electromobility shift assay shows that each receptor binds to a biotin-labeled ERE and migrates as expected (BfER, 521 amino acids, ∼58 kD; BfSR, 616 amino acids, ∼68 kD). When the two receptors are co-transfected, ERE-binding by the BfER homodimer is so strong that it is unclear whether BfER and BfSR heterodimerize. D) Mutation C205A, which compromises DNA binding, abrogates BfER's capacity to repress BfSR-mediated transcription of an ERE-driven reporter. Full-length BfSR, wild-type BfER (BfER-WT), and the BfER C205A mutant were cotransfected. Cells were treated with no hormone (white bars) or 1 µM estradiol (black bars).

In contrast, the BfER LBD has lost the ancestral capacity to activate transcription in response to estrogens. Neither the BfER LBD nor the full-length BfER activates transcription in response to high doses of any of a large panel of vertebrate steroid hormones, including estradiol, estriol, and estrone (Figures 3A, S5). In the absence of hormone, full-length BfER (like other estrogen receptors) represses basal levels of ERE-reporter transcription, but hormone treatment does not alter this effect (Figure 4A). On an SRE-driven reporter, full-length BfER has no effect (Figure 4B). BfER does retain the ancestral DNA-recognition function: a fusion construct of the BfER-DBD with the constitutively-active NFkB activation domain yields robust transcription of an ERE-driven reporter (Figure 5A), but no activity on an SRE-driven reporter (Figure 5B). Further, BfER strongly binds EREs in an electrophoretic mobility shift assay (EMSA, Figure 5C).

### BfER Is a Repressor of BfSR Activity

Because BfSR and BfER both interact with EREs, but only the former is activated by estrogens, we hypothesized that BfER might function as a competitive inhibitor of BfSR-activated transcription. To test this hypothesis, we cotransfected full-length BfER and BfSR in varying ratios with an ERE-driven reporter in the presence of estrogens. As predicted, BfER inhibited the transcriptional activity of BfSR in a manner dependent on the ratio of BfER to BfSR (Figure 4A). BfER's inhibitory activity is mediated by competition for EREs: alanine replacement of Cys^205^, a residue in the DNA-binding domain crucial for response element recognition (Figure 5A, see also [64]), abolishes BfER's capacity to repress BfSR-mediated transcription, even when transfected in very high ratios (Figure 5D, see also Figure 7C). In the EMSA, BfER binds very strongly to and competes for EREs as a homodimer; whether it can also heterodimerize with BfSR is unclear (Figure 5C). Taken together, these data indicate that competition for DNA is the primary mode by which BfER represses BfSR, although we cannot rule out a minor additional role for heterodimerization.

**Figure 6:**
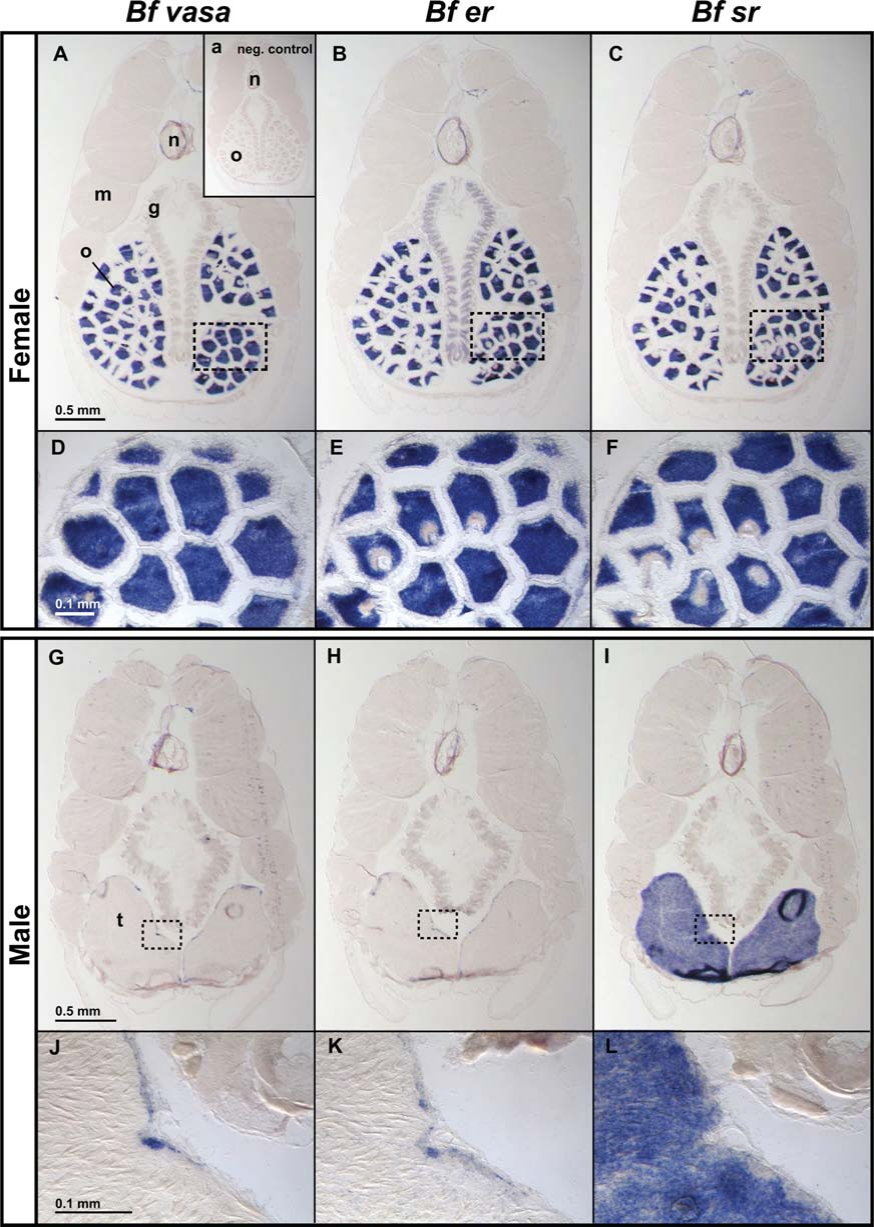
BfER and BfSR are co-expressed. Alternating cross-sections of adult animals were probed with digoxigenin-labelled RNAs for *Bfer*, *Bfsr*, or *Vasa*, a germ cell marker. A–F) In adult females, expression of *Bfer* and *Bfsr* are co-localized in the cytoplasm of oocytes in the ovary (o); *er* is also weakly expressed in the female gill bars (g). The negative control experiment with no probe (inset in *A*) shows no endogenous alkaline phosphatase activity. G–L) In adult males *Bfer and Vasa* are expressed only in a single cell layer of early spermatogonia in the testicular epithelium, but *Bfsr* is expressed throughout testes (t). Myotome (m) and notochord (n) are also labeled. Scale bars are indicated.

The hypothesis that BfER inhibits BfSR-mediated transcription predicts that the two genes should be coexpressed in some but not all tissues in vivo. We characterized the expression of BfER and BfSR in cross-sections of adult *B. floridae* by in situ hybridization. In females, transcripts of both genes are abundant in the cytoplasm of oocytes at multiple stages of differentiation (Figure 6, A–F) in a pattern identical to *Vasa*, a germ cell marker [80]. In addition, the *Bfer* transcript, but not *Bfsr*, is weakly expressed in female gills. In males (Figure 6, G–L), *Bfsr* is expressed broadly throughout the testes, in cells apparently at all stages of spermatogenesis. *Bfer*, in contrast, is expressed solely in the germinal epithelium of the testis, in a narrow band of cells that are likely to be early spermatogonia based on the expression of *Vasa* and their basal location within the testis [81]–[83]. These results are consistent with a role for the BfER and BfSR in regulating gonadal function. Because BfER suppresses the transcriptional activity of BfSR and these genes are coexpressed, we conclude that BfER functions, at least in part, as a competitive inhibitor of BfSR activity in vivo.

### Mechanisms of BfER Evolution

These results indicate that, after duplication of the estrogen-sensitive AncSR1, BfSR retained the ancestral function, whereas BfER evolved a new function as a repressor of the BfSR by losing its estrogen-activated transcriptional function but retaining its ability to compete for DNA response elements.

**Figure 7:**
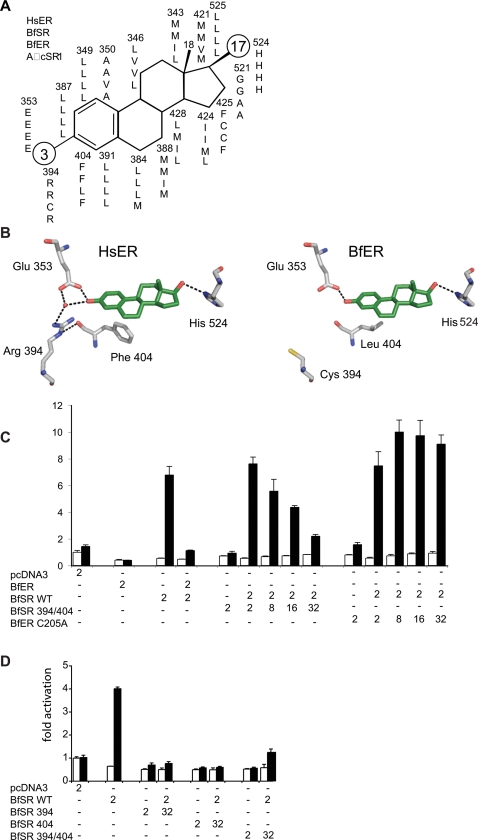
Mechanism for evolution of a duplicate receptor repressor. A) Conservation and variability of ligand-contacting residues. The figure shows a generic steroid hormone surrounded by the amino acids that line the ligand-binding pocket in the crystal structures of the human ERα. For each site, residues from the human ERα (HsER), BfSR, BfER, and AncSR1 are shown, from top to bottom, numbered according to the human ERα sequence. Moieties at the 3 and 17 positions, shown as large circles, vary among steroids. B) Historical substitutions R394C and F404L, which occurred in the lineage leading to transcriptional repressor BfER, are predicted to abolish estrogen binding and activation. Left, x-ray crystal structure of the ligand pocket of the human ERα with estradiol. Arg^394^ and Phe^404^ play key roles in a network of hydrogen bonds and packing interactions that stabilize the ligand and transcriptionally active conformation. Right, mutations R394C and F404L from BfER disrupt this network. Red sphere, water molecule. C) Mutations R394C and F404L, introduced into BfSR, abolish the receptor's transcriptional capacity and generate a dose-dependent repressor of the wild-type (WT) BfSR. Numbers show the quantity of each plasmid transfected (in ng) with 1 µM estradiol (black bars) or with no hormone added (vehicle only, white bars). Mutation C205A, which disrupts DNA-binding, eliminates BfER's capacity to repress BfSR-driven transcription. D) Either mutation R394C or F404L, introduced singly into BfSR, are each sufficient to abolish transcriptional capacity and generate a repressor of BfSR-WT.

To identify the mechanisms by which the ancestral ER was transformed into the repressor BfER, we compared the sequences of the BfER and BfSR LBDs to those of the human ERα and the reconstructed AncSR1 in light of existing structure-function information. Of the 18 residues that line the ligand-binding pocket of the human ERα [84], only three differ between BfSR and ERα. In contrast, 11 of 18 amino acids differ between the estrogen-insensitive BfER and the human ERα (Figure 7A). Examination of the crystal structures of human SRs predicts that two of the substitutions–R394C and F404L (numbered according to human the ERα sequence)–would have major effects on ligand binding and activation, because these residues, which are conserved in all vertebrate SRs, participate in a network of packing interactions and water-mediated hydrogen bonds that stabilize the receptor-ligand complex [85]. This network consists of a water molecule, Glu^353^, Arg^394^, Phe^404^, and the ligand's A-ring. Replacing Phe^404^ with a less bulky leucine would weaken the network's interaction with the ligand, and replacing the basic amino acid Arg^394^ with cysteine would abolish hydrogen bonds to both Phe^404^ and the water (Figure 7B). Both of these changes require a single nucleotide change and occurred in the BfER gene lineage, because the human ER-like states are also found in AncSR1 and all other steroid receptors sequenced to date, including BfSR.

To test the hypothesis that one or both of these historical replacements could have converted an estrogen-sensitive transcriptional activator into a competitive inhibitor, we introduced these changes into BfSR by site-directed mutagenesis. As predicted, the double-mutant BfSR R394C/F404L loses the wildtype's capacity to activate transcription in response to estrogens. Further, when co-transfected, the BfSR double-mutant repressed the activity of BfSR from an ERE in a concentration-dependent manner (Figure 7C). This repressive activity is dependent on ERE-binding and is not an artifact of co-transfection per se: unlike BfER and BfSR R394/F404L, co-transfecting increasing quantities of BfER-C205A, which does not compete for EREs, fails to repress BfSR-mediated reporter transcription (Figure 7C). Either mutation in isolation is sufficient to confer the full repressive phenotype on BfSR (Figure 7D). These data indicate that simple degenerative mutations, which abolish the protein's activation by estrogens but do not interfere with its ancestral DNA-binding activity, are sufficient to confer on BfER its novel function as a competitive inhibitor.

## Discussion

We found that BfSR, the *B. floridae* ortholog of the vertebrate kSRs, has molecular functions similar to those of the vertebrate estrogen receptor, whereas the cephalochordate ER ortholog is an estrogen-insensitive transcriptional repressor of BfSR-mediated transcription. The presence of estrogen-activated, ERE-recognizing receptors in both clades descending from AncSR1 corroborates previous findings that AncSR1 was ER-like in function [29],[66]. Our results make sense in light of the fact that cephalochordates and vertebrates diverged relatively recently after the duplication of AncSR1. In both taxa, one duplicate retained the ancestral function and the other evolved a new function, but the copy that experienced each fate differed between the two lineages. In the vertebrates, the ancestral kSR evolved novel ligand and response-element specificity, and ER retained the ancestral response to estrogen and EREs. In cephalochordates, the kSR ortholog retained the ancestral specificity for ligand and DNA, and the ER ortholog lost its estrogen-stimulated transcriptional activity, becoming a competitive repressor of BfSR on its DNA binding sites. BfER and BfSR have been recently recognized in the amphioxus genome sequence [50],[74]; this is the first characterization of their functions.

Our experiments shed light on the functions of steroid hormones and their receptors in cephalochordates. *B. floridae* produces estradiol [73], so it is likely that BfSR functions, at least in part, as a classic estrogen receptor. Although *B. floridae* also produces progesterone and testosterone, these steroids do not activate either receptor in this species, suggesting that they may function primarily as intermediates in the synthesis of estrogens or in other signaling pathways [29]. BfSR and BfER are strongly coexpressed in some but not all tissues, so it is likely that BfER functions as a BfSR regulator, presumably allowing finer tissue-specific modulation of BfSR activity. These receptors may also have other functions, such as activation by unknown cephalochordate-specific hormones or post-transcriptional modification [86],[87].

The expression of both BfER and BfSR primarily in testis and ovary suggests a role for both in gonadal function, such as regulating germ cell development and maturation. In females, both receptors are expressed strongly in ovary. In males, BfSR is expressed throughout the testis, including the apical zone near the testicular lumen, where later-stage spermatocytes, spermatids, and sperm are found. BfER, in contrast, is expressed only in the basal cells of the germinal epithelium, where early spermatogonia are located and where the germ-cell marker Vasa is also expressed [81]. In vertebrates, ERs also play important roles in testicular development and spermatogenesis, suggesting a conserved ancestral function [88],[89]. Further work in vivo is required to elucidate the physiological and developmental roles of BfER, BfSR, and estrogens in cephalochordates.

Our results demonstrate the strong effect that functional interactions can have on evolution after gene duplication and suggest the need to supplement existing models of this process. In both the classic and DDC models, degenerative mutations in one copy produce no change in phenotype. As a result, redundancy shields one copy from purifying selection, allowing high-probability degenerative mutations to accumulate neutrally; neofunctionalization must occur only through relatively low-probability gain-of-function mutations. Our results are not predicted by either model. Because steroid receptors function by interacting physically with other molecules, simple degenerative mutations in the BfER LBD produced a novel molecular phenotype–a protein that can no longer carry out the full functions of the ancestral gene but still competes for binding partners, thereby repressing the activity of its duplicate wherever the two are coexpressed. Evolutionary changes in the expression of BfSR and BfER also occurred, yielding partially overlapping expression domains. The order in which these coding and expression changes evolved, and their relative roles in the maintenance of the duplicated ancestral SR, cannot be resolved with current data.

The production of repressive functions by degenerative mutations may strongly affect the dynamics of evolution after gene duplication, particularly the relative probability of neofunctionalization versus gene loss by nonfunctionalization. If the repressor allele is adaptive — allowing beneficial new modes of regulating the other copy's activity–then it will be conserved, leading to long-term maintenance of both gene copies, as occurred with BfER and BfSR. This scenario points to a creative role for high-probability degenerative mutation not envisioned by any existing models. Because degenerative mutations are common, including them as a source of new functions would increase both the absolute and relative probability of neofunctionalization. We found that either of two historical point mutations in the BfER is sufficient to generate a repressor of BfSR; given the modular organization of steroid receptor domains, it is likely that there are many other potential mutations that could also have abolished estrogen-activation while leaving DNA-binding intact.

If, on the other hand, repression of the ancestral function is deleterious–as will often be the case–then purifying selection will tend to purge degenerative mutations from both copies, leading to conservation of both duplicates with the ancestral function. In such cases, the accumulation of degenerative mutation would be much slower than under the neutral scenarios of the classic and DDC models. In turn, the rate of nonfunctionalization would be slowed, and the temporal window during which neofunctionalization can occur would be lengthened substantially.

Evolution of beneficial duplicate repressors may be a widespread phenomenon. Numerous other members of the nuclear receptor (NR) superfamily have evolved by partial degeneration to function primarily as repressors of the transcriptional activity of paralogous NRs. Some have lost their capacity to activate transcription but retain their ability to bind DNA, so–like BfER–they compete for the binding sites of other receptors, whose activity they downregulate. Others have lost the capacity to bind DNA but retain the ability to form dimers with other NRs and thus inhibit their activity [90],[91]. Repressor duplicates have also evolved in beta-helix-loop-helix transcription factors: some duplicates have lost the canonical DNA-binding domain but still heterodimerize with and therefore silence their paralogs [47]. Similarly, in the family of transmembrane tumor necrosis factor receptors, which trigger apoptosis in response to extracellular ligands, primate-specific duplicates have evolved which bind ligand but have lost their capacity to interact with the intracellular factors that stimulate apoptosis. These “decoy receptors” compete for ligand and thereby repress the activity of their paralogs, preventing ligand-triggered apoptosis in cells that express receptors of both classes [92],[93].

As for the cases in which degenerative mutations are deleterious, it is not possible to directly determine the historical importance of selection against duplicate repressors in shaping genome evolution, but there are reasons to believe it may have played a significant role in delaying nonfunctionalization. Most protein families–enzymes, transcription factors, hormones, neurotransmitters, growth factors, to name a few–depend upon specific molecular interactions for their functions. In many of these families, separate domains, independent molecular surfaces, or even specific sets of residues mediate interactions with different partners or other aspects of function. It is therefore reasonable to expect that after duplication a large class of mutations would compromise specific aspects of function without abolishing all interactions, thereby producing competitive repressors. It is also likely that a nontrivial fraction of these alleles would be deleterious. If these assumptions are correct, then purifying selection would have played a role of general importance in purging degenerative mutations after gene duplication, maintaining duplicates for longer periods of time, and extending the temporal window during which neofunctionalization can occur. Indeed, it has been observed that duplicate genes involved in signal transduction have been preferentially retained in *Arabidopsis* [94]. This hypothesis also predicts that many genes after duplication will bear the mark of purifying selection–nonsynonymous-to-synonymous substitution rates considerably less than one–even in the absence of subfunctionalization. It has been observed that the majority of recent duplicate genes in *Paramecium* genomes are under strong purifying selection [46]. This signature is predicted to be particularly strong in duplicate genes that form homodimers or interact with partner molecules whose concentration is limiting for function, such as specific DNA binding sites.

Not all degenerative mutations in genes whose functions depend on interactions are likely to produce repressor alleles. Those that radically reduce expression, impair protein folding or stability, or increase the tendency for a protein to aggregate are expected to yield alleles compromised in their ability to interact with any and all partners. These fully nonfunctional alleles would have no effect on their duplicate's function and would be sheltered by redundancy as predicted by the classic and DDC models' neutral scenarios. Only those mutations that affect modular domains or molecular surfaces that control distinct subfunctions within the coding sequence have the potential to eliminate one aspect of a protein's functions without abolishing its interactions with at least some molecular partners. It is therefore likely that purifying selection would be partially relaxed after duplication–in contrast to the situation for unduplicated genes, in which all mutations that compromise function, including those that also generate novel functions, would be exposed to the constraining influence of selection. Ohno's idea that functional diversity can evolve by random exploration of sequence space after gene duplication may strain credulity in its original conception of a purely neutral setting and an inexorable tendency toward “entropic decay.”[95] It becomes far more plausible, however, when selection can play an anti-entropic role, and when creativity can arise not only from rare mutational combinations but also from far more common ones.

## Materials and Methods

### Receptor Isolation

Steroid receptor orthologs were identified in the *Branchiostoma floridae* genome database (Joint Genome Institute, v. 1.0) by tblastn search using as queries exons from the conserved DBD and LBD of human steroid receptors, as well as the inferred sequence of the ancestral steroid receptor [29],[66] and the ancestral corticosteroid receptor [96]. Recovered *B. floridae* sequences were used as queries to reciprocally search the human protein database using blastx, and those that recovered SR family members as best hits were retained. Using this technique, two *B. floridae* sequences were identified as likely SR orthologs.

From the two partial SR sequences recovered, primers were designed for RACE (Rapid Amplification of cDNA Ends). Amphioxus RNA was extracted from gravid individuals collected in October 2003 (Gulf Specimen Marine Lab, Panacea, FL) using the RNeasy kit (Qiagen, Valencia, CA). The isolated RNA was reverse transcribed using Thermoscript (Invitrogen, Carlsbad, CA) and oligo dT primers or PowerScript reverse transcriptase (Clontech, Mountain View, CA) with gene specific primers. Both 5′ and 3′ sequences were obtained by RACE using the SmartRace method (Clontech) and Phusion polymerase (New England BioLabs, Ipswich, MA). Products were cloned into TOPO TA cloning vector (Invitrogen) and multiple clones were sequenced (accessions EU371730 and 371729). Numerous synonymous polymorphisms were found in both receptor genes.

### Phylogenetic Analysis

The two amphioxus SR sequences were aligned to a database containing 140 other SR and closely related nuclear receptor protein sequences, including the *B. floridae* estrogen related receptor ERR (Table S1). The alignable portions of the amino acid sequences (DBD, LBD, conserved parts of the hinge, and CTE) were aligned using MUSCLE software [97]. Phylogenies (Figures 2B, S6, S7 and S8) were inferred using both Bayesian Markov-Chain Monte Carlo (BMCMC) and maximum likelihood (ML) methods. For BMCMC analysis, we used MrBayes v. 3.1 [98], using a search consisting of two independent runs of four chains each (one cold and three heated) of 4.8 million generations each, integration over protein models, gamma-distributed among-site rate variation (prior on the alpha parameter uniform (0.01 to 5), and uniform branch length priors (0,5]. Two million generations, a point well past stationarity (indicated as standard deviation of clade probabilities between runs of <0.015), were discarded as burn-in. The Jones-Taylor-Thornton model had 100% posterior probability in BMCMC analysis; this model and gamma-distributed rate variation were therefore used for ML analysis using PHYML-aLRT software; support was calculated as the approximate likelihood ratio and the chi-square confidence estimate derived from that likelihood ratio [99],[100].

Mixed-model maximum likelihood analysis was conducted using our SAML software [77], which calculates likelihoods as the weighted sum over heterogeneous branch length sets, with the number of sets defined by the user and the weight and branch length vector for each set estimated by maximum likelihood using a simulated annealing algorithm. Because of computational demands, a reduced dataset of 33 SR sequences was analyzed. For each model, 1000 perturbations were examined at 1000 temperatures from 1.0 to 0 with a setback of 10. For each proposal, the probability of topology rearrangement was 0.3 (of which 40%, 40%, and 20% were tree bisection/reconnection, subtree pruning/regrafting, and nearest-neighbor interchanges, respectively), of branch-length changes was 0.6, of changes in the alpha parameter of the gamma distribution was 0.1, and of the weight for each branch length set was 0.1. Analyses were conducted with models with from 1 to 6 branch length sets, and Akaike's Information Criterion was used to choose among models. The best-fit model had five categories and very high support (Aikake weight>0.999). Analysis with this model led to recovery of an ML phylogeny that also placed BfER sister to the clade of estrogen receptors and BfSR sister to the vertebrate AR/PR/GR/MR clade (Figure S3).

Synteny relationships were investigated using the human genome (NCBI v. 36, obtained from Ensembl v. 41) and the *B. floridae* genome (DOE Joint Genome Institute v. 1.0). To classify human proteins into paralogous groups, we used a single-linkage clustering algorithm based on reciprocal best hits in a BLASTp search of each protein in the human genome against all proteins in the human genome. To identify orthology relationships, BLASTp searches were performed between each human protein sequence and all proteins in the *B. floridae* genome, and between each *B. floridae* protein and all proteins in the human genome. Orthology relationships were inferred for reciprocal best hits between a human paralog group and a *B. floridae* protein. If multiple amphioxus proteins were reciprocal best hits for a human paralog group, the paralog group was split accordingly. A sliding window analysis was then performed between each human chromosome and all amphioxus scaffolds to group orthologs into conserved syntenic regions. A sliding window size of 100 genes was used. Figure 2C displays 19 ortholog pairs spanning ∼82 megabases of human chromosome 14 (77% of total chromosome length), ∼3 megabases of *B. floridae* scaffold 42 (100% of scaffold length), and 6 megabases of human chromosome 6 (3.5% of chromosome length). Non-orthologous genes that fall between regions of conserved synteny are not shown.

### Transcriptional Assays

Full-length amphioxus SR cDNAs were amplified using specific forward and reverse primers designed at start and stop codons and were cloned into the mammalian expression vector pcDNA3 (Invitrogen, Carlsbad, CA). Fusion constructs were prepared by amplifying the DBD (the canonical zinc finger region plus the first 30 amino acids in the hinge) or the LBD (including the hinge and carboxy-terminal extension) and subcloning them into pCMV-AD (Stratagene, La Jolla, CA) or pSG5-DBD (gift of D. Furlow), respectively. Site-directed mutagenesis was performed using QuickChange II (Stratagene, La Jolla, CA). All clones were verified by sequencing.

Reporter gene transcription assays were performed in Chinese Hamster Ovary (CHOK-1) cells grown to 90% confluence then harvested with trypsin (Invitrogen) and transferred to a 96-well plate containing phenol red-free αMEM supplemented with 10% dextran-charcoal-stripped fetal bovine serum and no antibiotics (Hyclone, Logan, UT). For LBD assays, cells were transfected using lipofectamine and Plus reagent (Invitrogen) with 1 ng receptor plasmid, 100 ng pFRluc reporter plasmid (Promega, Madison, WI), and 0.1 ng pRLtk reporter plasmid for normalization in Optimem (Invitrogen). For DBD assay, cells were transfected in Optimem with 4 ng receptor plasmid, 2 ng of reporter pGL3-4(EREc38)-luc (a firefly luciferase reporter driven by four estrogen response elements, a gift from C. Klinge) or SRE-luc (containing a TAT3 glucocorticoid response element and firefly luciferase ORF, a gift from B. Darimont), 0.1 ng normalization reporter pRLtk (Promega), and 95 ng of pUC19 DNA as filler for transfection efficiency. Four hours later, the transfection mixture was removed and replaced with antibiotic-free αMEM with 10% fetal bovine serum; cells were incubated overnight. LBD assays were then incubated with hormones or vehicle control in triplicate at each dose for an additional 24 hours. Cells were lysed and assayed for luminescence using Dual-Glo Luciferase System (Promega) on a Perkin-Elmer Victor3 plate reader. To calculate normalized luciferase activity, firefly luciferase luminescence was divided by Renilla luciferase luminescence. Dose-response relationships were calculated using nonlinear regression using Prism software (Graphpad, San Diego, CA).

Transcriptional activity of full-length receptors was assayed by transfecting CHO-K1 cells using reporter plasmids pGL3-4(EREc38)-luc or SRE-luc. Full length human GR in pcDNA3 (gift from B. Darimont) was used as the positive control on SREluc and treated with cortisol at a concentration of 10^−6^ M. Co-transfection of BfER and BfSR full-length plasmids was performed using identical conditions to individual full-length transcriptional assays except that cells were transfected with varying concentrations of each receptor (2 ng, 4 ng, 8 ng, 16 ng, or 32 ng per well), and treated with estradiol at 10^−6^ M. In screens of full-length receptor sensitivity to a broad panel of hormones, 5 ng receptor and 50 ng reporter were transfected. All experiments were done in triplicate and repeated at least twice with the same results.

### Electrophoretic Mobility Shift Assay

CHO-K1 cells were transfected with plasmids containing full-length BfER alone (4 µg), BfER (1 µg) and BfSR (4 µg), or BfSR alone (4 µg) as described above, treated with 1 µM estradiol for 4 hours and harvested in TEGDK buffer (10 mM Tris-HCL, 1 mM EDTA, 0.4 M KCL, 10% (vol/vol) glycerol, 1 mM dithiothreitol) with 1% protease inhibitor cocktail (Sigma-Aldrich, St. Louis, MO). Cells were lysed with four freeze-thaw cycles and centrifuged at 10,000×g for 20 min at 4 C. Protein was quantitated using the Bradford protein assay (Bio-Rad, Hercules, CA). Protein from BfER-transfected cells (2 µg), BfER+BFSR-transfected cells (5 µg), or BfSR-transfected cells (10 µg) was prepared and incubated with 10 ng biotinylated ERE- probe according to the manufacturer's protocol (Panomics, Redwood City, CA). Reactions were separated on a 5% native polyacrylamide gel in 1× tris-borate EDTA buffer for 3 hours at 90 V at 4 C. The long run was required to separate the sizes of BfER/ERE and BfSR/ERE complexes; in this time, unbound labelled probe migrated off the end of the gel. Gel contents were transferred to Biodyne B membrane (Pall, Ann Arbor, MI) for 30 min at 300 mA. Chemiluminescent detection of biotinylated DNA was performed using the Panomics EMSA kit.

### In Situ Hybridization

Gravid amphioxuswere collected in March, 2007 (Gulf Specimen Marine Lab, Panacea, FL) and fixed in 4% paraformaldehyde, 0.5 M NaCl, 1 mM MgSO4, 2 mM EGTA and 0.1 M MOPS. Fixed animals were dehydrated in a stepwise series of PBS∶methanol and stored in methanol at −20°C. Samples were re-hydrated in PBS, embedded in agar, and cross-sectioned in a cryostat at 16 mm. The germ-cell marker *vasa* was used as a positive control and to identify putative germ cells; *vasa* was identified in the *B. floridae* genome database using the human sequence as a query and amplified as described below. *B. floridae* cDNA fragments of BfER, BfSR, and BfVasa were amplified using the following primers: *er*-forward 5′- GCTAGTGCCTTTGACAAGTC -3′ and *er*-reverse 5′- CAGACACCTGGTCAGTGAG -3′ (corresponding to nucleotides 661–1501 of the BfER open reading frame), *sr*-forward 5′- AACTCATAGTGAGCCCCACC -3′ and *sr*-*reverse* 5′- CTGCAGAGTTCTCCACTGC -3′ (nucleotides 875–1724), and *vasa*-forward 5′- GAGCCAACGTGGCGAAGG -3′ and *vasa*-reverse 5′- CATCAGGGCCTCCTCTCTC -3′ (1–849 nt). The Vasa sequence has been deposited with accession EU371731. Amplicons were cloned into pCR4-TOPO vector (Invitrogen) and used to synthesize digoxigenin-labeled riboprobes (Boehringer-Mannheim). In situ hybridizations on cryo-sections were performed as described previously [101]. In situ hybridizations with different probes were performed on adjacent sections in alternate slides to facilitate comparison among the expression patterns of different genes. Negative control hybridizations were performed without riboprobe to identify any endogenous alkaline phosphatase activity.

## Supporting Information

Figure S1 Amino acid sequences of steroid receptor DNA-binding domains.
(0.31 MB PDF)

Figure S2 Amino acid sequences of steroid receptor ligand-binding domains.
(0.25 MB PDF)

Figure S3 Mixture-model maximum likelihood phylogenetic analysis.
(0.14 MB PDF)

Figure S4 Synteny analysis of BfER- and BfSR-containing scaffolds with human chromosomes.
(0.12 MB PDF)

Figure S5 Reporter expression assay of full-length BfER and BfSR.
(0.24 MB PDF)

Figure S6 Complete results of maximum likelihood phylogenetic analysis with likelihood ratio statistics.
(0.48 MB PDF)

Figure S7 Complete results of maximum likelihood phylogenetic analysis with chi-square support statistics.
(0.41 MB PDF)

Figure S8 Complete results of Bayesian phylogenetic analysis.
(0.40 MB PDF)

Table S1 Gene names, species, and accession numbers of sequences used in phylogenetic analyses.
(0.03 MB PDF)
