# Supplementary material for: Evolution of a New Function by Degenerative Mutation in Cephalochordate Steroid Receptors
Source: PLoS Genet. 2008 Sep 12;4(9):e1000191. doi: 10.1371/journal.pgen.1000191 (PMC2527136; doi:10.1371/journal.pgen.1000191)
Supplement: Figure S1 — Amino acid sequences of steroid receptor DNA-binding domains. (0.31 MB PDF) [file pgen.1000191.s001.pdf]

Figure S1

Amino acid sequence alignment of steroid receptor DNA-binding domains (DBD). The sequences fo cephalochordate receptors are aligned with DBDs of human steroid receptor family members. Mutation of residue C205A (boxed) in BfER prevents binding to DNA response elements.

DBD

```
                                     205
BfER         CRVCGDHASGFHYGVWSCEGCKAFFKRSIQQGQTDYICPGTNQCTIDRNRRKSCQACRYRKCLMVGM
BfSR         CAVCHCPSTGLHYGVYACEGCKSFFHRAHK-RAHPYVCPANNNCVIDRRLKKNCPACRLKKCLAMGM
HumanERα     CAVCNDYASGYHYGVWSCEGCKAFFKRSIQ-GHNDYMCPATNQCTIDKNRRKSCQACRLRKCYEVGM
HumanERβ     CAVCSDYASGYHYGVWSCEGCKAFFKRSIQ-GHNDYICPATNQCTIDKNRRKSCQACRLRKCYEVGM
HumanGR      CLVCSDEASGCHYGVLTCGSCKVFFKRAVE-GQHNYLCAGRNDCIIDKIRRKNCPACRYRKCLQAGM
HumanMR      CLVCGDEASGCHYGVVTCGSCKVFFKRAVE-GQHNYLCAGRNDCIIDKIRRKNCPACRLQKCLQAGM
HumanPR      CLICGDEASGCHYGVLTCGSCKVFFKRAME-GQHNYLCAGRNDCIVDKIRRKNCPACRLRKCCQAGM
HumanAR      CLICGDEASGCHYGALTCGSCKVFFKRAAE-GKQKYLCASRNDCTIDKFRRKNCPSCRLRKCYEAGM
```
