# Supplementary material for: Evolution of a New Function by Degenerative Mutation in Cephalochordate Steroid Receptors
Source: PLoS Genet. 2008 Sep 12;4(9):e1000191. doi: 10.1371/journal.pgen.1000191 (PMC2527136; doi:10.1371/journal.pgen.1000191)
Supplement: Figure S2 — Amino acid sequences of steroid receptor ligand-binding domains. (0.25 MB PDF) [file pgen.1000191.s002.pdf]

Figure S2
Amino acid sequence alignment of steroid receptor ligand-binding domains (LBD), with cephalochordate receptors aligned to LBDs of human steroid receptors. Mutations of hormone contacting residues R394C and F404L (boxed) eliminate estradiol activated transcription and generate a repressor of wild-type BfSR.

```
BfSR          PTLPLYNPTVPLISHLVNIEPNPILTGYNPQCTPTEGYLMALVTDLANREIEGLVDWAAR
BfER           LKALIDALDVKEGEHRGEENHPTGQQAGNWQEISNPELIESVSSLVDRELTGIICWGKK
HUMANERα          LSLTADQMVSALLDAEPP-ILYSEYDPTRPFSEASMMGLLTNLADRELVHMINWAKR
HumanERβ          DALSPEQLVLTLLEAEPPHVLISR--PSAPFTEASMMMSLTKLADKELVHMISWAKK
HumanGR               QLTPTLVSLLEVIEPEVLYAGYDSSVPDSTWRIMTTLNMLGGRQVIAAVKWAKA
HumanMR             RALTPSPVMVLENIEPEIVYAGYDSSKPDTAENLLSTLNRLAGKQMIQVVKWAKV
HumanPR         GQDIQLIPPLINLLMSIEPDVIYAGHDNTKPDTSSSLLTSLNQLGERQLLSVVKWSKS
HumanAR                CQPIFLNVLEAIEPGVVCAGHDNNQPDSFAALLSSLNELGERQLVHVVKWAKA

                                          394       404
BfSR          LPGYGMLPMDDQVNLIRTVWLDLLMLGLVW[R]SMEHRG-EWLV[F]APDLLMD-----RSLCR
BfER          IPGYSKLSLNDQVLLMESTWLDLLILDLVW[C]SIRHKG-EKLL[L]SGGVLVNRNTISNRRNN
HUMANERα      VPGFVDLTLHDQVHLLECAWLEILMIGLVW[R]SMEHPG--KLL[F]APNLLLDR----NQGKC
HumanERβ      IPGFVELSLFDQVRLLESCWMEVLMMGLMW[R]SIDHPG--KLI[F]APDLVLDR----DEGKC
HumanGR       IPGFRNLHLDDQMTLLQYSWMFLMAFALGW[R]SYRQSSANLLC[F]APDLIIN-----EQRMT
HumanMR       LPGFKNLPLEDQITLIQYSWMCLSSFALSW[R]SYKHTNSQFLY[F]APDLVFN-----EEKMH
HumanPR       LPGFRNLHIDDQITLIQYSWMSLMVFGLGW[R]SYKHVSGQMLY[F]APDLILN-----EQRMK
HumanAR       LPGFRNLHVDDQMAVIQYSWMGLMVFAMGW[R]SFTNVNSRMLY[F]APDLVFN-----EYRMH

BfSR          LSG----MEYICTPMLEFARQFADLQVPQEVYVCLKALTLYTTAV----SRLQDYRQVQR
BfER          SSGDDMEVLEMCDQILSIATKFYEFDLQRREYLCLKAITLVHG--------SLKGLESDT
HUMANERα      VEG----MVEIFDMLLATSSRFRMMNLQGEEFVCLKSIILLNSGVYTFLSSTLKSLEEKD
HumanERβ      VEG----ILEIFDMLLATTSRFRELKLQHKEYLCVKAMILLNSSMYPLVTATQDADSSR-
HumanGR       LPC----MYDQCKHMLYVSSELHRLQVSYEEYLCMKTLLLLS-------SVPKDGLKSQE
HumanMR       QSA----MYELCQGMHQISLQFVRLQLTFEEYTIMKVLLLLS-------TIPKDGLKSQA
HumanPR       ESS----FYSLCLTMWQIPQEFVKLQVSQEEFLCMKVLLLLN-------TIPLEGLRSQT
HumanAR       KSR----MYSQCVRMRHLSQEFGWLQITPQEFLCMKALLLFS-------IIPVDGLKNQK

BfSR          LQHEINEALAEACSSTFGFSPGNIARLMMIVSQVRQLSSLGVDHLNRLRGAETVSVEGLL
BfER          QVRQLQDDLTDALMDVCSER---HALGSRRPAKMLLL-------LSHLRQVSARASSHLG
HUMANERα      HIHRVLDKITDTLIHLMAKAGLTLQQQHQRLAQLLLI-------LSHIRHMSNKGMEHLY
HumanERβ      KLAHLLNAVTDALVWVIAKSGISSQQQSMRLANLLML-------LSHVRHASNKGMEHLL
HumanGR       LFDEIRMTYIKELGKAIVKREGNSSQNWQRFYQLTKL-------LDSMHEVVENLLNYCF
HumanMR       AFEEMRTNYIKELRKMVTKCPNNSGQSWQRFYQLTKL-------LDSMHDLVSDLLEFCF
HumanPR       QFEEMRSSYIRELIKAIGLRQKGVVSSSQRFYQLTKL-------LDNLHDLVKQLHLYCL
HumanAR       FFDELRMNYIKELDRIIACKRKNPTSCSRRFYQLTKL-------LDSVQPIARELHQFTF

BfSR          REIVDEPPRITEIEDSPGSAEGNKTMSRSAGKMECAASTSCTAQDEAVENSAEAEAEAAE
BfER          AVRNGLKVPLYDILLDILTDQVSEGQRDQQAHHEVASSPEKER-----------------
HUMANERα      SMKCKNVVPLYDLLLEMLDAHRLHAPTSRGGASVEETDQSHLATAGSTSSHSLQKYYITG
HumanERβ      NMKCKNVVPVYDLLLEMLNAHVLRGCKSSITGSECSPAEDSKSKEGSQNPQSQ-------
HumanGR       QTFLD-KTMSIEFPEMLAEIITNQIPKYSNGNIKKLLFHQ--------------------
HumanMR       YTFRESHALKVEFPAMLVEIISDQLPKVS-GNAKPLYFHRK-------------------
HumanPR       NTFIQSRALSVEFPEMMSEVIAAQLPKILAGMVKPLLFHKK-------------------
HumanAR       DLLIKSHMVSVDFPEMMAEIISVQVPKILSGKVKPIYFHTQ-------------------

BfSR          FAAQLKEVMEGEATTIADVKAMADQMGIEILDLD
BfER          ----------------------------------
HUMANERα      EAEGFPATV-------------------------
HumanERβ      ----------------------------------
HumanGR       ----------------------------------
HumanMR       ----------------------------------
HumanPR       ----------------------------------
HumanAR       ----------------------------------
```
