# Supplementary material for: Evolution of a New Function by Degenerative Mutation in Cephalochordate Steroid Receptors
Source: PLoS Genet. 2008 Sep 12;4(9):e1000191. doi: 10.1371/journal.pgen.1000191 (PMC2527136; doi:10.1371/journal.pgen.1000191)

Figure S3
Maximum likelihood phylogeny of a reduced steroid receptor dataset inferred using a mixed model that incorporates heterotachy (see methods).

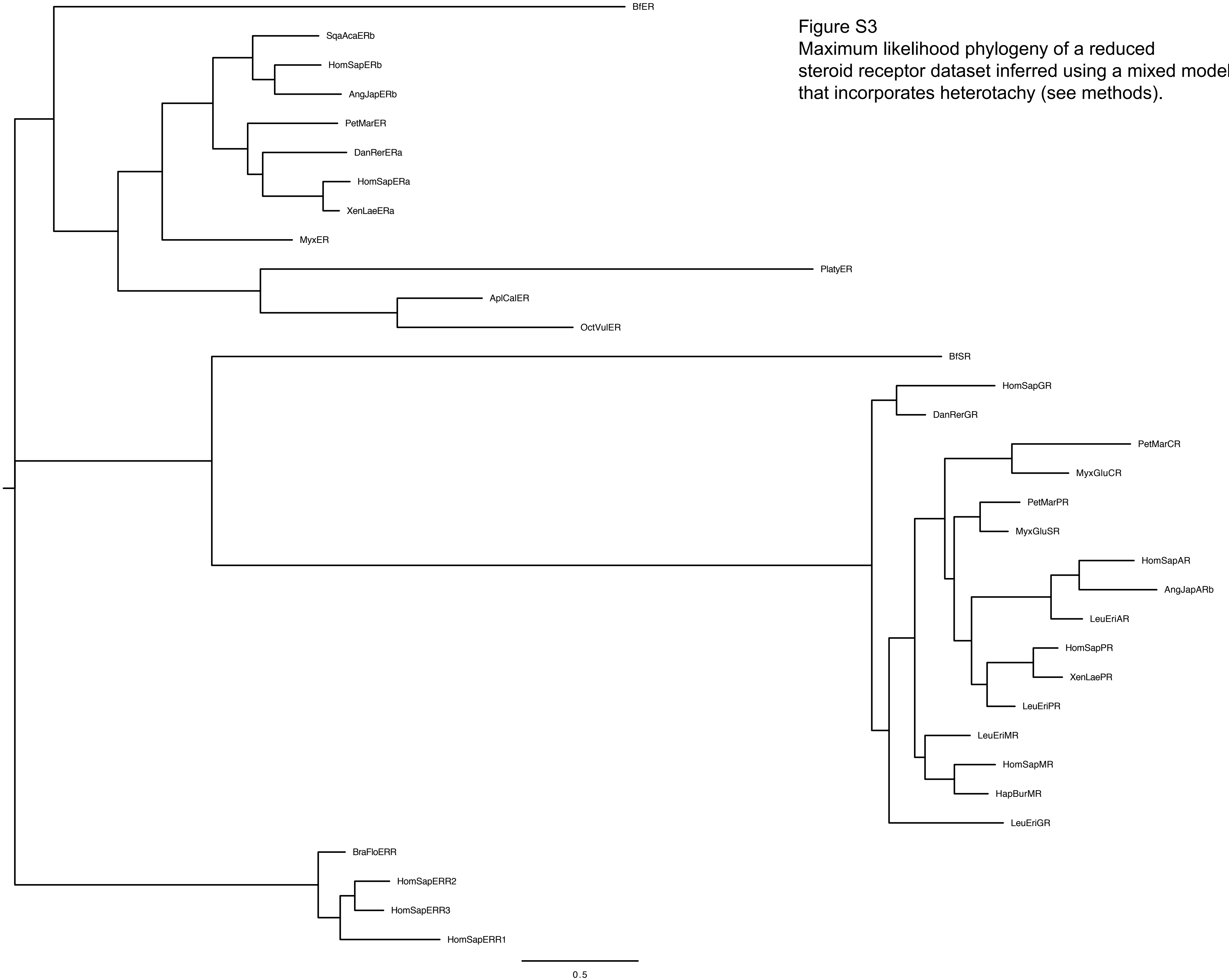

Supplement: Figure S3 — Mixture-model maximum likelihood phylogenetic analysis. (0.14 MB PDF) [file pgen.1000191.s003.pdf]
