# Supplementary material for: Evolution of a New Function by Degenerative Mutation in Cephalochordate Steroid Receptors
Source: PLoS Genet. 2008 Sep 12;4(9):e1000191. doi: 10.1371/journal.pgen.1000191 (PMC2527136; doi:10.1371/journal.pgen.1000191)

Fig. S4. Synteny analysis of scaffolds containing BfER and BfSR with the human genome. (A) Dotplot between B. floridae scaffold 42 (Bf42), containing BfER, and the human genome. Each gene on the amphioxus scaffold is shown along the horizontal axis as a grey dot. Human orthologs, if any, are marked directly above each B. floridae gene with a red cross on the appropriate human chromosome. This plot shows strong conserved synteny between human chromosome 14 and Bf42. (B) Dotplot between B. floridae scaffold 507 (Bf507), containing BfSR, and the human genome. The plot shows few B. floridae proteins on Bf507, few human orthologs, and no signal of conserved synteny between Bf507 and the human genome.

A

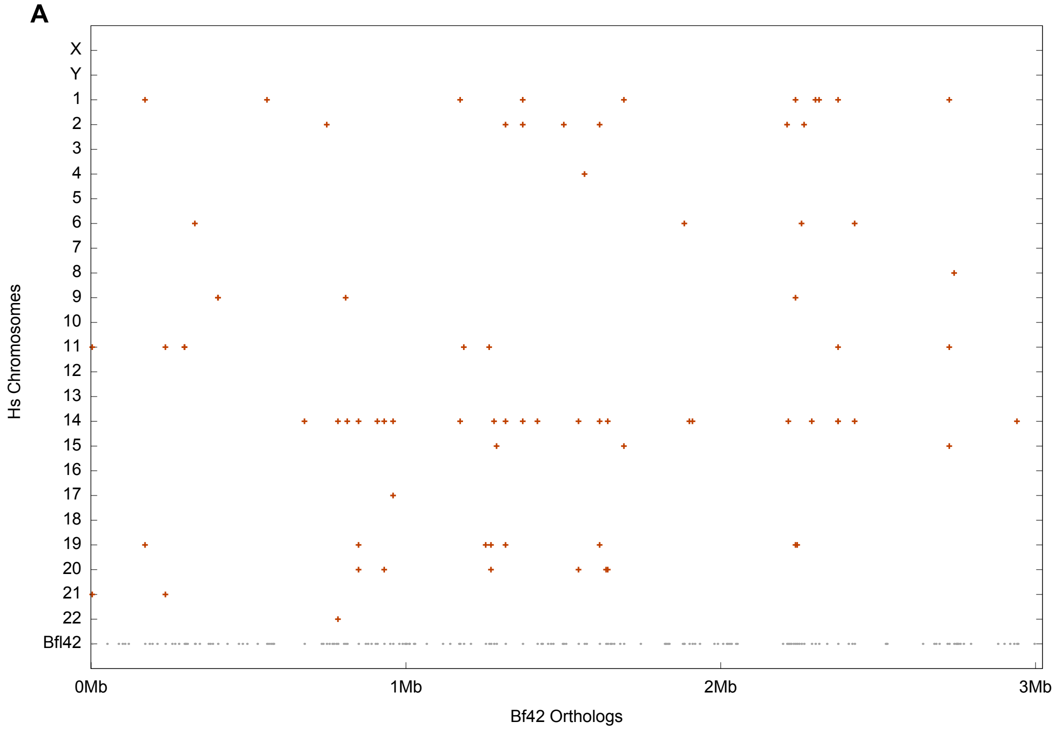

B

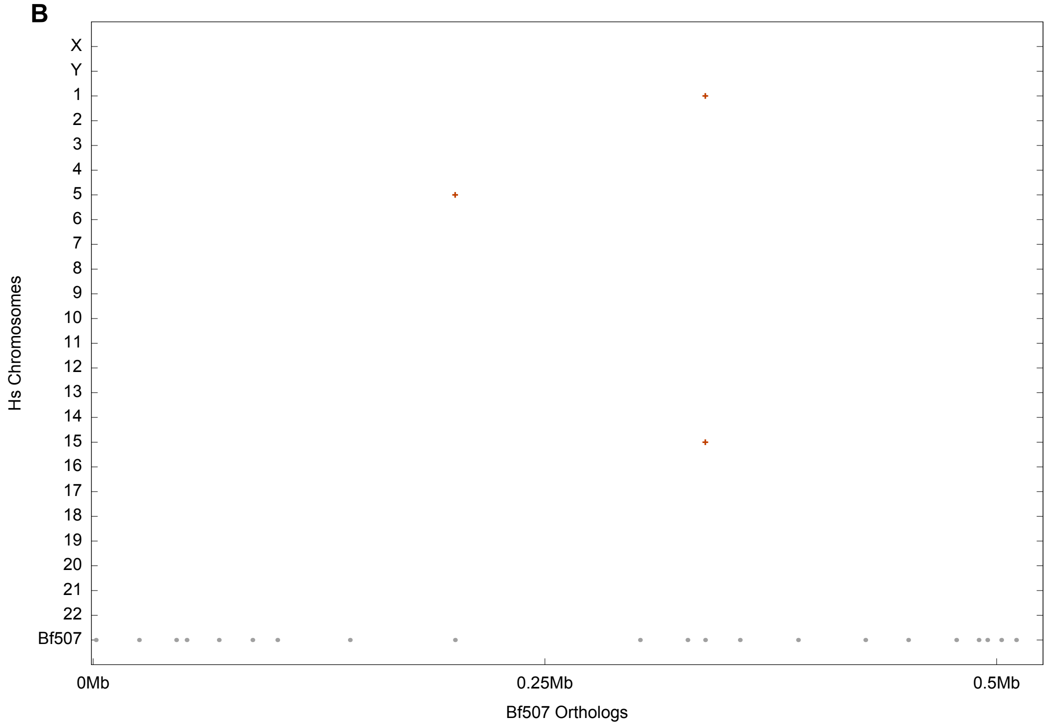

Supplement: Figure S4 — Synteny analysis of BfER- and BfSR-containing scaffolds with human chromosomes. (0.12 MB PDF) [file pgen.1000191.s004.pdf]
