# Supplementary material for: Evolution of a New Function by Degenerative Mutation in Cephalochordate Steroid Receptors
Source: PLoS Genet. 2008 Sep 12;4(9):e1000191. doi: 10.1371/journal.pgen.1000191 (PMC2527136; doi:10.1371/journal.pgen.1000191)

Figure S5

Hormone-activated transcription of a luciferase reporter gene by full-length BfSR (black) and BfER (gray) is shown as fold activation relative to vehicle-only control. CHOK-1 cells were transfected with 5ng of receptor along with 50ng of pGL3-4(EREc38)-luc reporter plasmid, 0.1ng pRLtk reporter plasmid for normalization, and 50ng PUC19 for transfection efficiency. All hormone treatments were at 1μM concentration. DES; diethylstilbesterol, DHT; dihydrotestosterone, DHEA; dehydroepiandrosterone. Mean +/- SE of three replicates is shown.

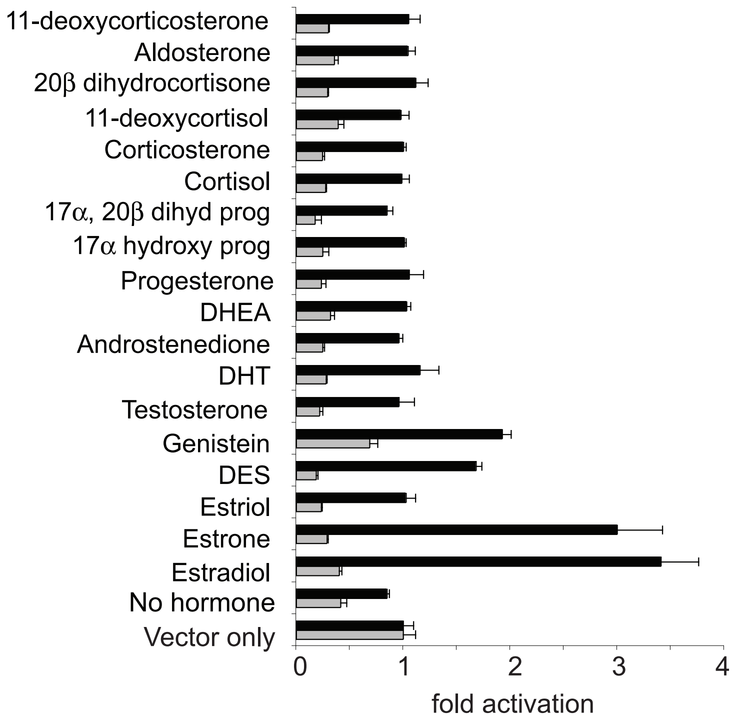

Supplement: Figure S5 — Reporter expression assay of full-length BfER and BfSR. (0.24 MB PDF) [file pgen.1000191.s005.pdf]
