# Supplementary material for: Evolution of a New Function by Degenerative Mutation in Cephalochordate Steroid Receptors
Source: PLoS Genet. 2008 Sep 12;4(9):e1000191. doi: 10.1371/journal.pgen.1000191 (PMC2527136; doi:10.1371/journal.pgen.1000191)

Figure S6
Maximum likelihood phylogeny with approximate likelihood ratio statistic (LRS) for each node calculated using PHYML software. LRS=2ln(L1/L2), where L1 is the likelihood of the maximum likelihood phylogeny and L2 is the likelihood of the most likely phylogeny lacking the labeled node.

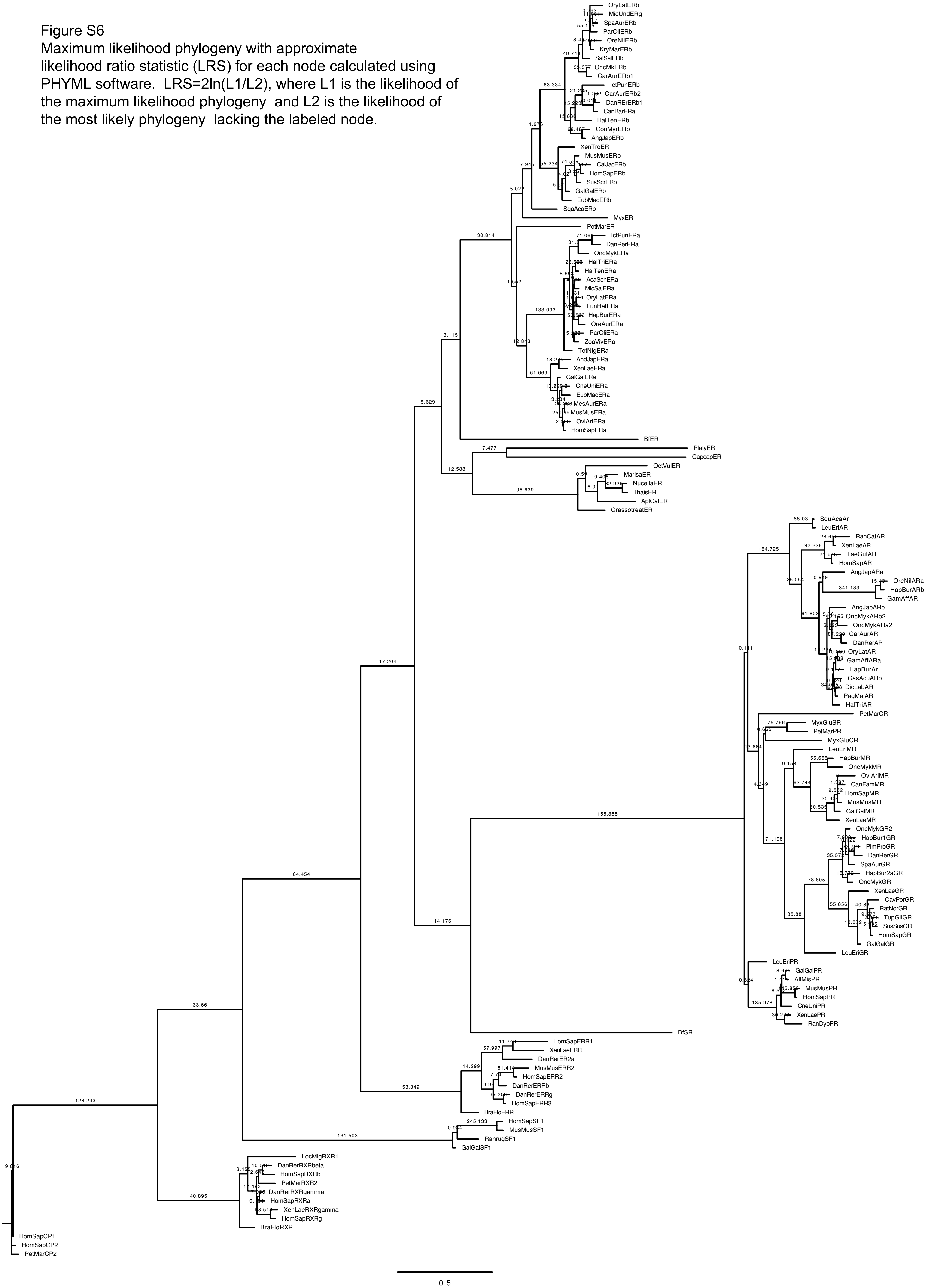

Supplement: Figure S6 — Complete results of maximum likelihood phylogenetic analysis with likelihood ratio statistics. (0.48 MB PDF) [file pgen.1000191.s006.pdf]
