# Supplementary material for: Evolution of a New Function by Degenerative Mutation in Cephalochordate Steroid Receptors
Source: PLoS Genet. 2008 Sep 12;4(9):e1000191. doi: 10.1371/journal.pgen.1000191 (PMC2527136; doi:10.1371/journal.pgen.1000191)

Figure S7

Maximum likelihood steroid receptor phylogeny with chi-square support statistics for each node. Support statistics were calculated using PHYML software based on the approximate likelihood ratio for each node. The chi-square support represents 1 - p, where p is the estimated probability that the degree of support observed for the labeled node would occur by chance if the node is false.

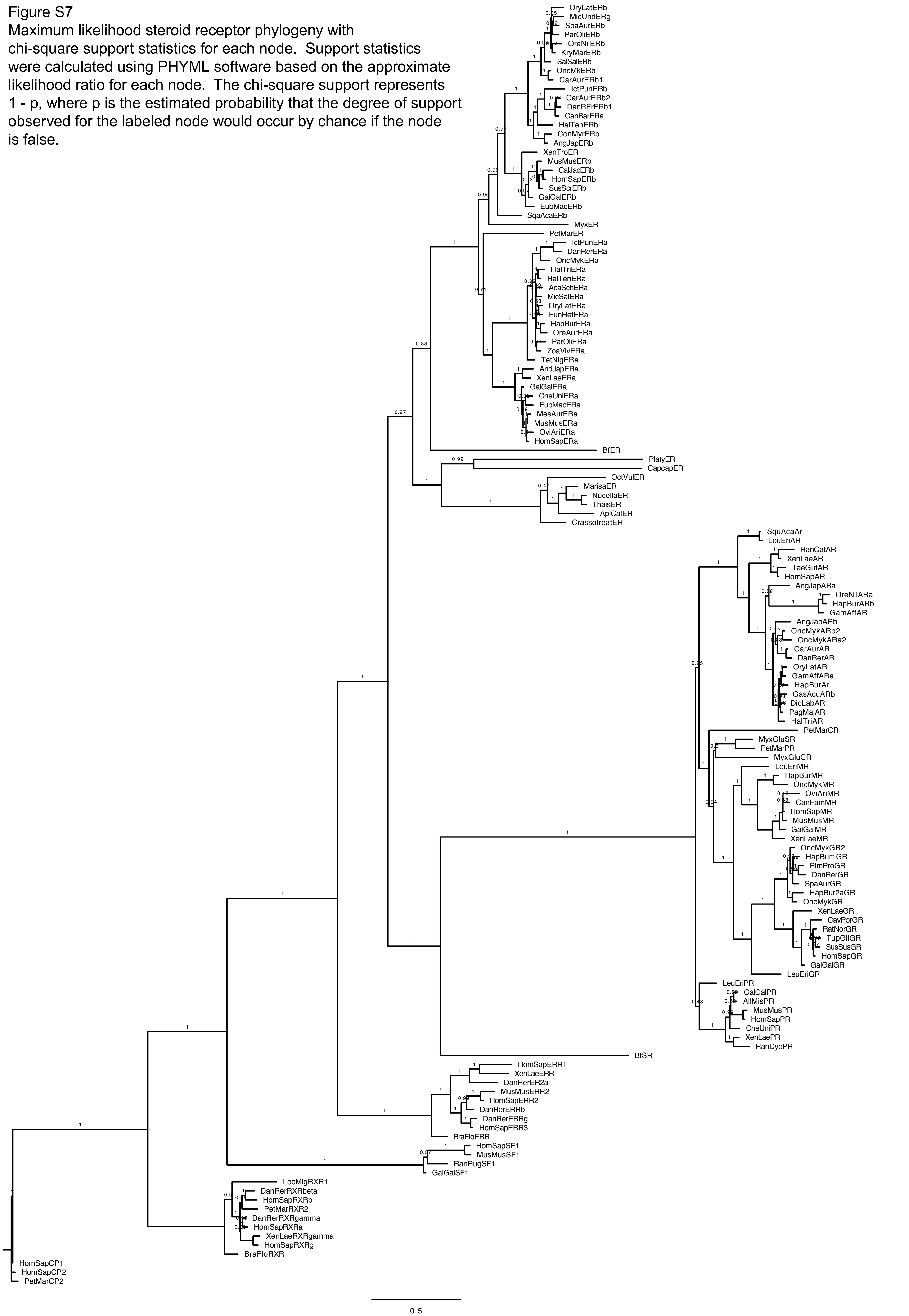

Supplement: Figure S7 — Complete results of maximum likelihood phylogenetic analysis with chi-square support statistics. (0.41 MB PDF) [file pgen.1000191.s007.pdf]
