# Supplementary material for: Evolution of a New Function by Degenerative Mutation in Cephalochordate Steroid Receptors
Source: PLoS Genet. 2008 Sep 12;4(9):e1000191. doi: 10.1371/journal.pgen.1000191 (PMC2527136; doi:10.1371/journal.pgen.1000191)
Supplement: Figure S8 — Complete results of Bayesian phylogenetic analysis. (0.40 MB PDF) [file pgen.1000191.s008.pdf]

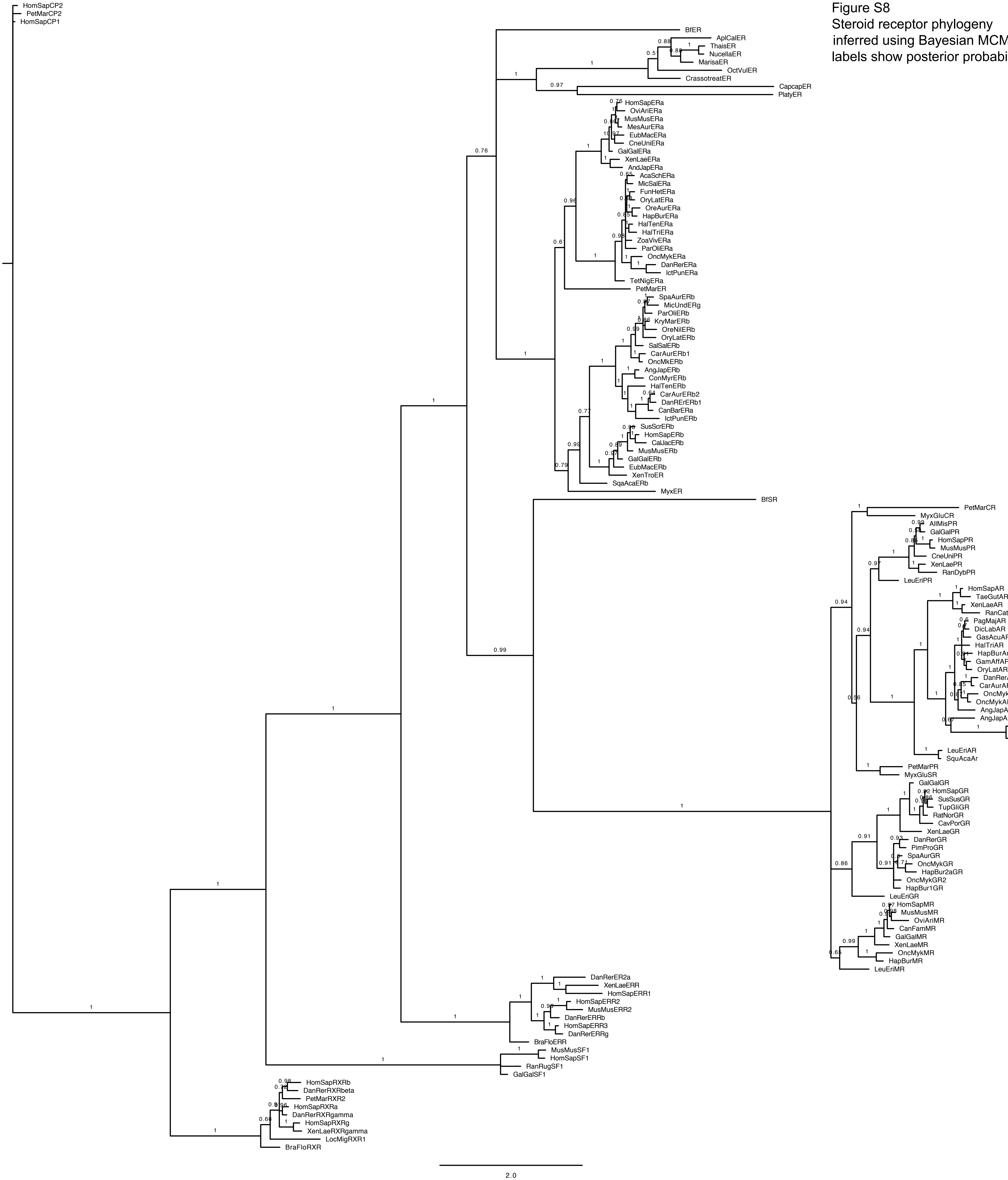

Figure S8
Steroid receptor phylogeny inferred using Bayesian MCMC. Node labels show posterior probabilities (>50%).
