# Supplementary material for: Evolution of a New Function by Degenerative Mutation in Cephalochordate Steroid Receptors
Source: PLoS Genet. 2008 Sep 12;4(9):e1000191. doi: 10.1371/journal.pgen.1000191 (PMC2527136; doi:10.1371/journal.pgen.1000191)
Supplement: Table S1 — Gene names, species, and accession numbers of sequences used in phylogenetic analyses. (0.03 MB PDF) [file pgen.1000191.s009.pdf]

Table S1 Sequences, species and accession numbers used in receptor phylogenies.

Sequence name	Species name	Common Name	Genbank or SwissProt accession#
AcaSchERa	Acanthopagrus schlegeli	black porgy	Q804Q6
AllMisPR	Alligator mississippiensis	American alligator	Q765N5
AndJapERa	Andrias japonicus	salamander	Q0KKA1
AngJapARa	Anguilla japonica	Japanese eel	Q9YGV9
AngJapARb	Anguilla japonica	Japanese eel	Q9PWG5
AngJapERb	Anguilla japonica	Japanese eel	BAA19851
AplCalER	Aplysia californica	sea slug	Q6VU64
BfER	Branchiostoma floridae	Florida amphioxus	EU371730
BfSR	Branchiostoma floridae	Florida amphioxus	EU371729
BraFloERR	Branchiostoma floridae	Florida amphioxus	AAU88063
BraFloRXR	Branchiostoma floridae	Florida amphioxus	Q8MX78
CalJacERb	Callithrix jacchus	common marmoset	Q95171
CanBarERa	Candidia barbatus	Taiwanese cyprinid fish	Q7T3U5
CanFamMR	Canis familiaris	dog	XP_532685
CapcapER	Capitella capitata	polychaete worm	EU497681
CarAurAR	Carassius auratus	goldfish	Q8QFV2
CarAurERb1	Carassius auratus	goldfish	Q9W669
CarAurERb2	Carassius auratus	goldfish	Q9IAL9
CavPorGR	Cavia porcellus	guinea pig	P49115
CneUniERa	Cnemidophorus uniparens	whip-tailed lizard	Q8UWA9
CneUniPR	Cnemidophorus uniparens	whip-tailed lizard	Q91425
ConMyrERb	Conger myriaster	conger eel	Q762D6
CrassotreatER	Crassotrea gigas	pacific oyster	BAF45381
DanRerAR	Danio rerio	zebrafish	Q6EAL5
DanRerER2a	Danio rerio	zebrafish	Q7ZU32
DanRerERa	Danio rerio	zebrafish	P57717
DanRErERb1	Danio rerio	zebrafish	Q90WS9
DanRerERRb	Danio rerio	zebrafish	Q6Q6F5
DanRerERRg	Danio rerio	zebrafish	Q6Q6F4
DanRerGR	Danio rerio	zebrafish	NP_001018547
DanRerRXRbeta	Danio rerio	zebrafish	Q7SYN5
DanRerRXRgamn	Danio rerio	zebrafish	18859345
DicLabAR	Dicentrarchus labrax	European sea bass	Q4G497
EubMacERa	Eublepharis macularius	leopard gecko	Q2HWK5
EubMacERb	Eublepharis macularius	leopard gecko	Q2HWK4
FunHetERa	Fundulus heteroclitus	killifish	Q7SZI0
GalGalERa	Gallus gallus	chicken	P06212
GalGalERb	Gallus gallus	chicken	Q9PTU5
GalGalGR	Gallus gallus	chicken	Q38HX7

GalGalMR	Gallus gallus	chicken	XP_420437
GalGalPR	Gallus gallus	chicken	P07812
GalGalSF1	Gallus gallus	chicken	Q9PW17
GamAffAR	Gambusia affinis	western mosquitofish	Q60I32
GamAffARa	Gambusia affinis	western mosquitofish	Q60GT9
GasAcuARb	Gasterosteus aculeatus	three-spined stickelback	Q801Z2
HalTenERa	Halichoeres tenuispinis	wrasse	Q7T2K8
HalTenERb	Halichoeres tenuispinis	wrasse	Q7T2K7
HalTriAR	Halichoeres trimaculatus	protogynous wrasse	Q9DDJ4
HalTriERa	Halichoeres trimaculatus	protogynous wrasse	Q9DDJ3
HapBur1GR	Haplochromis burtoni	African cichlid	Q8JJ92
HapBur2aGR	Haplochromis burtoni	African cichlid	Q8JJ91
HapBurAr	Haplochromis burtoni	African cichlid	Q9W6F4
HapBurARb	Haplochromis burtoni	African cichlid	Q8QFV7
HapBurERa	Haplochromis burtoni	African cichlid	Q6TGB3
HapBurMR	Haplochromis burtoni	African cichlid	Q8JJ89
HomSapAR	Homo sapiens	human	P10275
HomSapCP1	Homo sapiens	human	P10589
HomSapCP2	Homo sapiens	human	P24468
HomSapERa	Homo sapiens	human	P03372
HomSapERb	Homo sapiens	human	Q92731
HomSapERR1	Homo sapiens	human	P11474
HomSapERR2	Homo sapiens	human	O95718
HomSapERR3	Homo sapiens	human	P26508
HomSapGR	Homo sapiens	human	P04150
HomSapMR	Homo sapiens	human	P08235
HomSapPR	Homo sapiens	human	P06401
HomSapRXRa	Homo sapiens	human	P1979
HomSapRXRb	Homo sapiens	human	P28702
HomSapRXRg	Homo sapiens	human	P48443
HomSapSF1	Homo sapiens	human	Q13285
IctPunERa	Ictalurus punctatus	channel catfish	Q9YHZ7
IctPunERb	Ictalurus punctatus	channel catfish	Q9IAK1
KryMarERb	Kryptolebias marmoratus	mangrove rivulus	Q0KKB9
LeuEriAR	Leucoraja erinacea	little skate	Q1KXY2
LeuEriGR	Leucoraja erinacea	little skate	Q1KXY4
LeuEriMR	Leucoraja erinacea	little skate	Q1KXY3
LeuEriPR	Leucoraja erinacea	little skate	Q1KXY1
LocMigRXR1	Locusta migratoria	locust	Q6V7U7
MarisaER	Marisa cornuarietis	freshwater snail	ABI97119
MesAurERa	Mesocricetus auratus	golden hamster	Q9QZJ5
MicSalERa	Micropterus salmoides	largemouth bass	Q9DDZ4
MicUndERg	Micropogonias undulatus	Atlantic croaker	P57783

MusMusERa	Mus musculus	mouse	P19785
MusMusERb	Mus musculus	mouse	O08537
MusMusERR2	Mus musculus	mouse	Q61539
MusMusMR	Mus musculus	mouse	Q8VII8
MusMusPR	Mus musculus	mouse	Q00175
MusMusSF1	Mus musculus	mouse	P33242
MyxER	Myxine glutinosa	hagfish	EU439936
MyxGluCR	Myxine glutinosa	hagfish	ABD46742
MyxGluSR	Myxine glutinosa	hagfish	ABD46743
NucellaER	Nucella lapillus	gastropod	ABQ96884
OctVulER	Octopus vulgaris	octopus	Q19AB0
OncMkERb	Oncorhynchus mykiss	rainbow trout	P57782
OncMykARa2	Oncorhynchus mykiss	rainbow trout	O93244
OncMykARb2	Oncorhynchus mykiss	rainbow trout	O93245
OncMykERa	Oncorhynchus mykiss	rainbow trout	P16058
OncMykGR	Oncorhynchus mykiss	rainbow trout	P49843
OncMykGR2	Oncorhynchus mykiss	rainbow trout	Q6RKQ3
OncMykMR	Oncorhynchus mykiss	rainbow trout	Q5WP02
OreAurERa	Oreochromis aureus	Israel tilapia	P50240
OreNilARa	Orechromis niloticus	Nile tilapia	Q8UWB8
OreNilERb	Orechromis niloticus	Nile tilapia	Q7T3U4
OryLatAR	Oryzias latipes	Japanese ricefish	Q76LM5
OryLatERa	Oryzias latipes	Japanese ricefish	P50241
OryLatERb	Oryzias latipes	Japanese ricefish	Q8UW75
OviAriERa	Ovis aries	sheep	Q95L13
OviAriMR	Ovis aries	sheep	Q9BDJ7
PagMajAR	Pagrus major	red sea bream	O93497
ParOliERa	Paralichthys olivaceus	Japanses flounder	Q8QHL0
ParOliERb	Paralichthys olivaceus	Japanses flounder	Q8QHK9
PetMarCP2	Petromyzon marinus	sea lamprey	Q801J3
PetMarCR	Petromyzon marinus	sea lamprey	AAK20930
PetMarER	Petromyzon marinus	sea lamprey	AAK20929
PetMarPR	Petromyzon marinus	sea lamprey	AAK20931
PetMarRXR2	Petromyzon marinus	sea lamprey	Q2PK06
PimProGR	Pimephales promelas	fathead minnow	Q2TU59
PlatyER	Platynereis dumerilii	annelid	EU482033
RanCatAR	Rana catesbeiana	bullfrog	Q7T1K4
RanDybPR	Rana dybowskii	Korean brown frog	Q8AYI2
RanRugSF1	Rana rugosa	frog	BAA36789
RatNorGR	Rattus norvegicus	rat	P06536
SalSalERb	Salmo salar	Atlantic salmon	Q6R7S4
SpaAurERb	Sparus aurata	gilthead seabream	Q9W6M2
SpaAurGR	Sparus aurata	gilthead seabream	Q1HGK6

SqaAcaERb	Squalus acanthias	spiny dogfish	Q90ZE6
SquAcaAr	Squalus acanthias	spiny dogfish	AAP55843
SusScrERb	Sus scrofa	pig	Q9XSW2
SusSusGR	Sus scrofa	pig	Q9N1U3
TaeGutAR	Taeniopygia guttata	zebrafinch	Q2VP86
TetNigERa	Tetraodon nigroviridis	green-spotted pufferfish	CAG03596
ThaisER	Thais clavigera	marine snail	BAC66480
TupGliGR	Tupaia glis	common tree shrew	Q95267
XenLaeAR	Xenopus laevis	African clawed toad	P70048
XenLaeERa	Xenopus laevis	African clawed toad	P81559
XenLaeERR	Xenopus laevis	African clawed toad	Q6AX97
XenLaeGR	Xenopus laevis	African clawed toad	P49844
XenLaeMR	Xenopus laevis	African clawed toad	Q66J29
XenLaePR	Xenopus laevis	African clawed toad	Q9DDU9
XenLaeRXRgamn	Xenopus laevis	African clawed toad	P51129
XenTroER	Xenopus tropicalis	clawed toad	Q6W5G8
ZoaVivERa	Zoarces viviparus	Eelpout (fish)	Q800Q2
